# Supplementary material for: Multi-omic signatures identify pan-cancer classes of tumors beyond tissue of origin
Source: Sci Rep. 2020 May 20;10:8341. doi: 10.1038/s41598-020-65119-5 (PMC7239905; doi:10.1038/s41598-020-65119-5)
Supplement: Supplementary file 1 — Supplementary information. [file 41598_2020_65119_MOESM1_ESM.pdf]

Multi-omic signatures identify pan-cancer classes of tumors beyond tissue of origin.

Agustin Gonzalez-Reymundez[1], Ana I. Vazquez[1*].

[1]Department of Epidemiology and Biostatistics, Michigan State University, MI, USA.

[1]Institute for Quantitative Health Science and Engineering (IQ), Michigan State University, MI, USA.

*avazquez@msu.edu

Table S1: Complete list of genes significantly de-regulated in at least one pan-cancer cluster. All the genes significantly different in at least one cluster are sorted by chromosome (**Chr**) and genomic position (in base pairs). Significant differences between clusters are the results of Tukey tests on the enrichment scores for each gene (computed as the linear combination of omic features mapping onto the gene, and their corresponding loadings). Different letters represented with different letters according to . Genes highlighted with red represent known cancer-related genes.

	Enrichment scores by cluster														
**Gene**	**C1**	**C2**	**C3**	**C4**	**C5**	**C6**	**C7**	**C8**	**Chr**	**Start**	**End**	**GE**	**METH**	**CNV**	**p_value**
C1orf159	0.02 b	0.10 ab	-0.07 b	0.15 ab	0.21 ab	0.41 a	0.00 b	-0.09 b	1	1081818	1116361	NA	-0.04	NA	6.36E-13
WASH2P	-0.29 b	0.21 a	0.00 b	0.05 ab	0.17 ab	-0.16 b	-0.04 b	0.05 ab	2	113588550	113599043	0.00	NA	NA	9.78E-14
RAB6C	0.08 ab	-0.04 bc	-0.01 b	-0.09 bc	-0.42 bc	0.43 a	-0.12 bc	-0.48 c	2	129979664	129982738	0.03	NA	NA	4.47E-17
ITM2C	0.07 ab	0.01 b	-0.06 b	0.15 ab	0.02 ab	0.41 a	0.07 ab	-0.07 b	2	230864639	230879248	-0.02	NA	NA	4.94E-11
HSP90AB2P	-0.19 ab	0.02 ab	0.05 ab	0.28 a	0.12 ab	-0.03 ab	-0.51 b	-0.16 ab	4	13333414	13338657	-0.03	NA	NA	2.62E-15
MCC	-0.11 c	-0.03 bc	-0.08 c	0.42 ab	0.22 abc	0.60 a	0.26 abc	0.29 abc	5	113022099	113488830	NA	-0.03	NA	1.35E-33
STK38	-0.23 ab	-0.14 ab	0.09 a	-0.26 ab	-0.14 ab	0.12 a	-0.38 b	-0.01 ab	6	36493892	36547470	0.05	NA	-0.01	1.92E-18
PPIL1	-0.11 ab	-0.10 ab	0.09 a	-0.23 ab	-0.27 ab	-0.04 ab	-0.44 b	-0.12 ab	6	36854827	36875024	0.02	NA	-0.01	3.22E-15
C6orf89	0.07 ab	0.10 a	-0.03 ab	0.05 ab	0.19 a	0.05 ab	-0.33 ab	0.14 a	6	36871870	36928964	0.05	NA	-0.01	5.86E-06
MTCH1	-0.09 ab	0.08 a	0.00 ab	0.12 a	0.06 ab	0.11 a	-0.45 b	0.08 ab	6	36968141	36986298	-0.08	NA	-0.01	5.02E-09
TBC1D22B	-0.03 a	0.06 a	0.03 a	-0.05 ab	-0.15 ab	-0.03 a	-0.50 b	0.07 a	6	37257772	37332970	0.04	NA	-0.01	2.49E-09
RNF8	-0.15 ab	0.00 a	0.05 a	-0.19 ab	-0.14 ab	0.13 a	-0.46 b	-0.01 ab	6	37353972	37394738	0.04	NA	-0.01	1.05E-11

CMTR1	-0.03 ab	-0.02 a	0.05 a	-0.04 ab	-0.10 ab	-0.06 ab	-0.46 b	-0.02 ab	6	37433219	37482827	0.04	NA	-0.01	1.40E-08
ZFAND3	-0.04 ab	0.06 a	0.02 ab	0.05 ab	0.14 a	-0.09 ab	-0.43 ab	-0.03 ab	6	37819499	38154624	-0.02	NA	-0.01	1.92E-07
BTBD9	-0.03 a	0.13 a	0.03 a	0.29 a	-0.10 a	0.05 a	-1.19 b	0.03 a	6	38168451	38640148	0.04	-0.01	-0.02	1.04E-61
GLO1	-0.21 ab	0.07 a	0.05 a	0.00 ab	0.02 ab	-0.08 ab	-0.50 b	-0.09 ab	6	38675925	38703141	0.04	NA	-0.02	1.67E-14
SAYSD1	-0.17 ab	0.01 a	0.04 a	0.17 a	0.21 a	-0.02 a	-0.48 ab	0.18 a	6	39104064	39115189	0.00	NA	-0.02	1.60E-12
LRFN2	-0.02 b	0.12 ab	0.05 b	0.49 a	-0.14 b	0.05 b	-1.60 c	0.05 b	6	40391586	40587465	NA	0.05	-0.03	1.10E-118
UNC5CL	0.02 ab	-0.05 ab	0.01 ab	0.00 ab	0.02 ab	0.18 a	-0.36 b	0.10 ab	6	41026911	41039217	-0.03	NA	-0.03	5.13E-06
OARD1	0.18 a	0.13 a	-0.03 a	0.19 a	0.21 a	0.17 a	-0.87 b	0.01 a	6	41033627	41097787	0.06	NA	-0.03	1.16E-37
NFYA	-0.09 a	0.09 a	0.04 a	0.06 a	-0.14 ab	-0.04 a	-0.77 b	0.05 a	6	41072945	41099976	0.02	NA	-0.03	4.39E-25
FOXP4	0.02 a	0.07 a	0.01 a	0.27 a	0.27 a	-0.04 a	-0.67 b	0.10 a	6	41546426	41602384	0.04	NA	-0.03	1.26E-19
TFEB	0.16 ab	0.09 ab	-0.07 ab	0.34 a	0.31 ab	0.31 ab	-0.31 b	0.15 ab	6	41683978	41736259	-0.09	NA	-0.03	2.71E-18
FRS3	-0.01 b	0.16 b	0.05 b	0.76 a	0.02 b	0.06 b	-2.04 c	0.13 b	6	41770176	41786542	-0.05	0.12	-0.04	9.81E-207
PRICKLE4	0.13 a	0.06 a	-0.03 a	0.02 a	-0.07 a	0.13 a	-0.29 a	0.13 a	6	41780762	41787372	0.02	NA	-0.04	1.07E-05
TOMM6	-0.11 a	0.03 a	0.08 a	0.32 a	-0.13 a	0.02 a	-1.24 b	-0.03 a	6	41787662	41789898	-0.04	NA	-0.04	7.35E-70
USP49	-0.01 a	0.03 a	0.04 a	0.00 a	-0.05 ab	-0.11 ab	-0.65 b	0.06 a	6	41789896	41895361	0.01	NA	-0.04	2.95E-17
MED20	-0.05 a	0.02 a	0.09 a	0.15 a	-0.06 a	-0.08 a	-1.28 b	-0.06 a	6	41905354	41921139	0.05	NA	-0.04	2.09E-72
BYSL	-0.17 a	-0.03 a	0.10 a	0.19 a	-0.28 ab	0.00 a	-1.09 b	-0.18 a	6	41921188	41933046	0.03	NA	-0.04	3.39E-58
CCND3	-0.01 a	0.14 a	-0.02 a	0.40 a	0.12 a	0.16 a	-0.77 b	0.09 a	6	41934933	42050357	-0.05	NA	-0.04	1.24E-30
TAF8	0.08 a	0.12 a	0.00 a	0.29 a	0.02 a	0.10 a	-1.06 b	0.16 a	6	42050513	42087461	0.01	NA	-0.04	2.53E-50
GUCA1A	-0.03 b	0.15 b	0.06 b	0.91 a	0.00 b	0.05 b	-2.16 c	0.04 b	6	42155406	42180056	NA	-0.04	-0.04	5.46E-242
GUCA1B	0.06 ab	0.10 ab	-0.04 ab	0.36 a	-0.12 ab	0.06 ab	-0.29 b	0.13 ab	6	42184401	42194916	0.02	NA	-0.04	3.81E-08
MRPS10	-0.11 a	0.03 a	0.08 a	0.14 a	-0.18 a	0.02 a	-1.22 b	-0.15 a	6	42206801	42217865	0.01	NA	-0.04	5.96E-67
TRERF1	-0.01 b	0.15 b	0.05 b	0.92 a	-0.03 b	0.10 b	-2.05 c	0.09 b	6	42224931	42452051	0.07	0.08	-0.04	6.77E-218

UBR2	0.03 a	0.11 a	0.03 a	0.22 a	0.11 a	-0.11 a	-0.94 b	0.06 a	6	42564062	42693504	0.03	NA	-0.04	4.27E-38
TBCC	-0.08 a	0.06 a	0.04 a	0.40 a	-0.05 a	0.09 a	-1.01 b	0.04 a	6	42744481	42746096	0.00	NA	-0.04	2.47E-46
BICRAL	0.10 a	0.13 a	-0.02 a	0.28 a	0.32 a	-0.10 a	-0.60 b	0.16 a	6	42746958	42868560	0.00	NA	-0.04	9.26E-20
RPL7L1	-0.10 a	0.09 a	0.04 a	0.29 a	0.08 a	-0.01 a	-1.01 b	0.04 a	6	42879618	42889925	0.02	NA	-0.04	2.92E-45
C6orf226	0.07 a	0.04 a	0.00 a	0.29 a	-0.02 ab	0.07 a	-0.68 b	0.22 a	6	42890265	42890816	0.08	NA	-0.04	2.97E-20
CNPY3	0.06 ab	0.09 ab	-0.01 b	0.54 a	0.28 ab	0.12 ab	-0.96 c	0.05 ab	6	42929192	42939287	-0.07	NA	-0.04	3.29E-46
GNMT	0.09 abc	0.26 ab	-0.10 c	0.48 a	0.18 abc	0.02 abc	-0.09 bc	0.21 abc	6	42960758	42963880	0.00	NA	-0.04	3.01E-22
PEX6	0.03 a	0.15 a	-0.02 a	0.34 a	-0.04 ab	0.06 a	-0.64 b	0.07 a	6	42963870	42979220	0.02	NA	-0.04	1.66E-21
PPP2R5D	-0.08 a	0.09 a	0.05 a	0.32 a	-0.03 a	0.00 a	-1.18 b	-0.05 a	6	42984499	43012342	0.05	NA	-0.04	2.00E-61
MEA1	-0.08 a	0.05 a	0.06 a	0.36 a	-0.08 a	-0.01 a	-1.22 b	0.00 a	6	43012094	43013968	0.04	NA	-0.04	3.13E-66
KLHDC3	-0.02 a	0.12 a	0.02 a	0.43 a	-0.10 a	0.17 a	-1.14 b	-0.04 a	6	43014103	43021298	0.04	NA	-0.04	3.64E-60
RRP36	-0.07 a	0.06 a	0.06 a	0.30 a	0.02 a	0.02 a	-1.18 b	-0.03 a	6	43021645	43034156	0.06	NA	-0.04	2.84E-61
CUL7	-0.01 a	0.05 a	0.04 a	0.25 a	0.05 a	-0.12 a	-0.92 b	0.20 a	6	43037617	43053945	0.04	NA	-0.04	6.30E-37
KLC4	-0.02 a	0.06 a	0.00 a	0.39 a	0.28 a	0.25 a	-0.86 b	0.15 a	6	43040777	43075099	0.02	NA	-0.04	3.94E-37
MRPL2	-0.08 a	-0.01 a	0.06 a	0.39 a	-0.29 a	0.17 a	-1.15 b	-0.03 a	6	43054029	43059806	-0.02	NA	-0.04	2.73E-61
PTK7	0.00 a	0.01 a	0.02 a	0.18 a	-0.28 ab	0.09 a	-0.56 b	0.05 a	6	43076268	43161719	-0.06	NA	-0.04	1.39E-12
SRF	0.11 a	0.09 a	-0.02 a	0.30 a	0.26 a	0.15 a	-0.75 b	0.06 a	6	43171299	43181507	0.03	NA	-0.04	6.28E-27
CUL9	0.09 a	0.08 a	0.01 a	0.33 a	-0.03 a	-0.01 a	-0.89 b	0.07 a	6	43182175	43224587	-0.08	NA	-0.04	1.75E-34
DNPH1	0.02 a	-0.05 a	0.02 a	0.33 a	-0.12 ab	0.21 a	-0.74 b	0.28 a	6	43225629	43229484	0.08	NA	-0.04	1.48E-27
CRIP3	0.16 ab	0.07 ab	-0.08 ab	0.30 a	0.25 ab	0.29 a	-0.12 ab	0.34 a	6	43299710	43308797	0.08	NA	-0.04	2.50E-16
ZNF318	0.00 a	0.08 a	0.04 a	0.16 a	0.08 a	-0.11 a	-0.85 b	0.02 a	6	43307134	43369478	-0.06	NA	-0.04	3.81E-30
ABCC10	-0.13 a	-0.02 a	0.07 a	0.25 a	0.00 a	-0.02 a	-0.90 b	0.05 a	6	43427366	43450430	0.04	NA	-0.04	4.20E-37
TJAP1	-0.07 a	0.12 a	0.05 a	0.36 a	-0.26 a	-0.04 a	-1.15 b	-0.04 a	6	43477523	43506556	0.07	NA	-0.04	1.10E-60

LRRC73	0.09 ab	0.02 ab	-0.01 ab	0.38 a	0.22 ab	-0.11 ab	-0.35 b	0.11 ab	6	43506969	43510686	0.01	NA	-0.04	1.32E-08
POLR1C	-0.12 a	0.00 a	0.06 a	0.37 a	-0.02 a	0.09 a	-1.15 b	0.09 a	6	43509702	43529585	0.03	NA	-0.04	3.73E-60
YIPF3	-0.01 b	0.11 ab	0.02 b	0.47 a	0.21 ab	0.04 ab	-1.09 c	0.06 ab	6	43511827	43516990	-0.09	NA	-0.04	9.49E-55
XPO5	-0.17 a	-0.07 a	0.12 a	0.18 a	-0.11 a	-0.14 a	-1.15 b	-0.08 a	6	43522330	43576075	-0.08	NA	-0.04	9.67E-68
POLH	-0.01 b	0.16 b	0.06 b	0.94 a	-0.02 b	0.04 b	-2.14 c	0.06 b	6	43576150	43615660	0.01	-0.39	-0.04	4.63E-239
GTPBP2	-0.16 b	-0.01 ab	0.10 a	0.06 ab	-0.35 bc	-0.02 ab	-1.00 c	-0.11 ab	6	43605316	43629162	0.00	NA	-0.04	4.93E-48
MAD2L1BP	-0.09 a	0.09 a	0.05 a	0.32 a	0.02 a	0.10 a	-1.11 b	-0.15 a	6	43629540	43640952	0.04	NA	-0.04	1.38E-55
MRPS18A	0.02 a	0.01 a	0.04 a	0.46 a	-0.07 a	0.11 a	-1.14 b	-0.04 a	6	43671303	43687791	0.02	NA	-0.04	3.73E-59
VEGFA	-0.16 ab	0.00 ab	0.04 ab	0.14 a	0.07 ab	0.05 ab	-0.50 b	-0.03 ab	6	43770184	43786487	0.02	NA	-0.04	6.75E-12
MRPL14	-0.16 b	0.04 ab	0.05 ab	0.37 a	-0.25 bc	0.16 ab	-0.98 c	0.00 ab	6	44113454	44127457	0.03	NA	-0.04	8.68E-47
TMEM63B	-0.04 a	0.06 a	0.07 a	0.20 a	-0.35 ab	-0.16 a	-1.01 b	-0.21 a	6	44126914	44155519	-0.09	NA	-0.04	1.97E-48
CAPN11	0.13 ab	0.15 ab	-0.07 b	0.42 a	-0.07 ab	0.03 ab	-0.09 b	0.25 ab	6	44158811	44184402	0.02	NA	-0.04	3.67E-12
SLC29A1	0.00 a	-0.02 a	0.06 a	0.14 a	-0.30 ab	-0.10 a	-0.74 b	0.08 a	6	44219505	44234151	0.06	NA	-0.04	6.62E-24
HSP90AB1	-0.19 a	0.03 a	0.10 a	0.30 a	-0.18 a	-0.15 a	-1.13 b	-0.19 a	6	44246166	44253888	0.00	NA	-0.04	1.20E-65
SLC35B2	-0.09 a	0.01 a	0.05 a	0.33 a	-0.06 a	0.09 a	-1.12 b	0.15 a	6	44254096	44257890	0.04	NA	-0.04	7.46E-56
NFKBIE	-0.06 ab	0.00 a	0.01 a	0.25 a	-0.04 ab	0.21 a	-0.64 b	0.22 a	6	44258166	44265788	-0.09	NA	-0.04	5.80E-20
AARS2	-0.06 a	0.02 a	0.05 a	0.33 a	-0.25 ab	0.07 a	-1.04 b	-0.02 a	6	44299654	44313326	-0.07	NA	-0.04	8.22E-48
CDC5L	-0.07 a	0.08 a	0.04 a	0.25 a	0.03 a	0.04 a	-1.06 b	-0.06 a	6	44387525	44450426	0.00	NA	-0.04	1.54E-48
SUPT3H	0.12 a	0.04 a	0.00 a	0.22 a	0.20 a	0.06 a	-0.70 b	0.04 a	6	44809317	45377953	0.02	NA	-0.04	7.20E-21
SLC25A27	-0.05 b	0.00 b	-0.03 b	0.23 ab	0.23 ab	0.34 a	-0.11 b	0.11 ab	6	46652915	46678193	0.02	NA	-0.04	1.55E-08
TDRD6	-0.02 b	0.09 b	0.05 b	1.29 a	-0.12 b	-0.06 b	-1.72 c	-0.06 b	6	46687875	46704319	-0.02	0.08	-0.04	1.46E-176
CD2AP	-0.05 ab	0.04 a	0.03 a	0.23 a	0.02 ab	-0.04 ab	-0.68 b	0.11 a	6	47477789	47627263	-0.09	NA	-0.04	3.75E-19
MUT	-0.03 ab	0.08 a	0.01 a	0.39 a	-0.01 ab	0.01 a	-0.57 b	-0.01 ab	6	49430360	49463191	0.07	NA	-0.04	1.43E-15

CENPQ	-0.21 a	-0.05 a	0.10 a	0.10 a	-0.21 ab	-0.06 a	-0.69 b	-0.26 ab	6	49463378	49493107	0.01	NA	-0.04	1.37E-29
MCM3	-0.16 a	-0.05 a	0.10 a	0.21 a	-0.20 ab	-0.07 a	-0.81 b	-0.28 ab	6	52264009	52284881	0.01	NA	-0.04	3.84E-36
PAQR8	-0.08 b	0.16 b	0.02 b	1.52 a	-0.11 b	0.06 b	-1.79 c	0.02 b	6	52361421	52407777	0.07	0.25	-0.04	1.29E-209
EFHC1	0.05 ab	0.04 ab	-0.01 ab	0.35 a	0.29 ab	0.02 ab	-0.52 b	0.16 ab	6	52362123	52529886	0.06	NA	-0.04	2.00E-13
TRAM2	0.05 abc	0.10 ab	-0.01 bc	0.45 a	0.08 abc	-0.07 bc	-0.56 c	0.08 abc	6	52497402	52576915	0.00	NA	-0.04	1.72E-17
TMEM14A	-0.08 ab	-0.03 ab	0.09 a	0.28 a	-0.14 abc	-0.15 abc	-0.79 c	-0.36 bc	6	52671109	52686588	0.04	NA	-0.04	1.78E-35
GSTA4	0.11 ab	0.08 ab	-0.02 ab	0.30 a	0.34 a	-0.11 ab	-0.42 ab	0.11 ab	6	52977948	52995378	-0.03	NA	-0.04	1.99E-11
ICK	-0.02 ab	0.10 ab	0.02 ab	0.44 a	0.23 ab	-0.25 bc	-0.66 c	0.04 ab	6	53001279	53061802	-0.03	NA	-0.04	4.90E-26
FBXO9	0.02 bc	0.10 bc	-0.02 bc	0.61 a	0.13 abc	-0.06 bc	-0.55 c	0.13 ab	6	53051991	53100873	0.02	NA	-0.04	1.42E-21
GCM1	-0.12 a	-0.06 a	0.03 a	0.35 a	-0.06 a	-0.10 a	-0.17 a	0.11 a	6	53126964	53148829	0.01	NA	-0.04	1.37E-05
ELOVL5	0.04 ab	0.11 ab	-0.02 b	0.51 a	0.28 ab	-0.20 b	-0.28 b	0.03 ab	6	53267398	53349179	0.04	NA	-0.04	1.81E-12
GCLC	0.02 ab	-0.01 ab	0.06 ab	0.21 a	0.06 ab	-0.26 ab	-0.70 b	-0.12 ab	6	53497341	53616970	0.02	NA	-0.04	1.37E-25
KLHL31	-0.03 ab	0.01 ab	0.02 ab	0.32 a	-0.02 ab	-0.14 ab	-0.36 b	0.13 ab	6	53647901	53665708	0.00	NA	-0.04	5.98E-08
LRRC1	-0.03 a	0.02 a	0.05 a	0.14 a	-0.13 ab	-0.24 ab	-0.59 b	0.08 a	6	53794497	53924121	0.01	NA	-0.04	2.03E-17
DST	0.09 a	0.06 a	0.02 ab	0.03 ab	-0.10 ab	-0.13 ab	-0.40 b	-0.18 ab	6	56457987	56954628	0.00	NA	-0.03	4.53E-08
KIAA1586	-0.06 ab	0.08 ab	0.01 ab	0.19 a	0.12 ab	0.00 ab	-0.58 b	0.16 a	6	57046532	57055239	0.04	NA	-0.03	2.59E-14
ZNF451	-0.04 a	0.10 a	0.01 a	0.36 a	-0.12 ab	-0.03 a	-0.55 b	0.07 a	6	57086844	57170307	-0.04	NA	-0.03	6.26E-15
BAG2	0.06 ab	0.11 ab	-0.06 b	0.17 ab	0.08 ab	0.24 a	-0.16 b	0.13 ab	6	57172326	57189833	0.00	NA	-0.03	4.47E-08
PRIM2	-0.18 ab	0.05 a	0.05 a	0.21 a	-0.05 ab	-0.05 ab	-0.53 b	-0.16 ab	6	57314805	57646849	0.02	NA	-0.03	1.93E-15
GUSBP4	-0.03 a	0.02 a	0.03 a	0.27 a	-0.07 ab	-0.03 a	-0.57 b	0.00 a	6	57919784	57930291	-0.02	NA	-0.03	1.83E-13
PHF3	0.01 a	0.13 a	-0.02 a	0.01 a	0.35 a	-0.06 a	-0.24 a	0.14 a	6	63635820	63779336	0.07	NA	-0.01	2.09E-05
THSD7A	0.04 ab	0.20 a	-0.13 ab	0.42 a	0.55 a	0.35 a	0.20 ab	0.18 ab	7	11370357	11832198	NA	-0.01	NA	1.35E-31
INTS4P2	0.00 b	0.00 b	-0.05 b	0.13 ab	-0.15 b	0.58 a	-0.01 b	-0.23 b	7	65647823	65718688	0.00	NA	NA	6.07E-21

ARHGEF10	-0.17 a	0.06 a	-0.03 a	0.14 a	0.38 a	0.20 a	0.15 a	0.03 a	8	1823976	1958641	NA	-0.01	NA	1.05E-06
HTRA4	-0.17 b	-0.07 ab	0.03 ab	-0.10 ab	-0.09 ab	0.23 a	-0.11 ab	-0.14 ab	8	38974164	38988662	NA	-0.01	NA	2.05E-06
DIP2C	0.02 ab	-0.01 b	-0.02 b	-0.12 b	-0.01 b	0.35 a	-0.04 b	-0.13 b	10	274190	689668	NA	-0.02	NA	1.46E-06
DNA2	0.02 b	0.00 b	-0.06 b	0.00 b	0.06 ab	0.55 a	0.06 b	0.03 b	10	68414064	68472121	NA	-0.02	NA	7.72E-18
HKDC1	-0.06 b	-0.07 b	-0.04 b	0.06 ab	0.03 ab	0.55 a	0.11 ab	0.23 ab	10	69220303	69267559	0.03	NA	NA	4.08E-20
CTBP2	0.10 b	0.08 b	-0.10 b	0.13 ab	0.08 b	0.51 a	0.17 ab	0.09 b	10	124984317	125161170	NA	NA	NA	5.97E-23
FAM196A	-0.04 b	-0.10 b	0.02 b	-0.09 b	0.04 ab	0.39 a	-0.21 b	-0.33 b	10	127135426	127196158	0.06	NA	NA	9.00E-14
CSNK2A3	-0.51 c	0.38 a	-0.08 bc	0.23 ab	0.18 abc	0.22 ab	0.20 ab	0.21 ab	11	11351942	11353357	0.00	NA	NA	8.41E-53
FOLH1	0.06 a	0.14 a	-0.06 a	0.31 a	0.28 a	0.10 a	-0.13 a	0.10 a	11	49146635	49208670	0.06	NA	NA	6.03E-08
MRPL16	-0.12 b	-0.13 b	0.00 b	-0.13 b	0.01 ab	0.50 a	0.00 b	0.09 ab	11	59806135	59810872	0.03	NA	0.00	8.72E-17
EIF1AD	0.23 ab	0.07 ab	-0.09 b	0.27 ab	0.02 ab	0.28 a	0.18 ab	-0.21 b	11	65996545	66002176	0.00	NA	0.01	5.57E-17
SF3B2	0.11 a	-0.03 a	-0.03 a	0.21 a	-0.20 a	0.21 a	0.07 a	-0.27 a	11	66050729	66069308	0.02	NA	0.01	4.31E-06
PACS1	-0.11 b	-0.07 b	0.01 b	0.06 ab	-0.15 b	0.32 a	0.02 ab	-0.26 b	11	66070363	66244747	0.03	NA	0.02	4.39E-08
KLC2	0.11 b	-0.03 b	-0.06 b	0.15 ab	-0.06 b	0.58 a	0.05 b	-0.18 b	11	66257294	66267860	0.07	NA	0.02	1.75E-22
RAB1B	0.04 b	-0.08 b	0.00 b	0.06 ab	-0.29 b	0.42 a	-0.06 b	-0.40 b	11	66268533	66277492	0.05	NA	0.02	5.73E-14
YIF1A	0.17 ab	0.07 ab	-0.05 bc	0.00 abc	-0.03 abc	0.31 a	0.04 abc	-0.42 c	11	66284580	66289170	0.01	NA	0.02	3.28E-13
BRMS1	0.15 ab	0.05 ab	-0.07 b	0.16 ab	0.03 ab	0.38 a	0.06 ab	-0.22 b	11	66337333	66345125	0.03	NA	0.03	1.47E-13
MRPL11	0.16 ab	0.10 ab	-0.07 b	0.19 ab	0.00 ab	0.28 a	0.06 ab	-0.16 b	11	66435075	66466738	0.02	NA	0.03	4.33E-11
PELI3	-0.02 b	-0.06 b	-0.03 b	0.06 b	-0.23 b	0.60 a	0.06 b	-0.34 b	11	66466327	66477337	-0.04	NA	0.03	1.59E-23
DPP3	0.17 b	0.09 b	-0.10 b	0.19 ab	-0.19 b	0.54 a	0.06 b	-0.17 b	11	66480013	66509657	0.07	NA	0.03	2.36E-27
BBS1	-0.12 b	-0.09 b	0.04 ab	0.06 ab	-0.51 b	0.28 a	-0.07 ab	-0.43 b	11	66510606	66533627	0.03	NA	0.03	1.69E-12
ZDHHC24	0.08 b	0.04 b	-0.05 bc	-0.03 bc	-0.23 bc	0.63 a	-0.04 bc	-0.52 c	11	66520637	66546238	0.04	NA	0.03	1.25E-30
CCDC87	-0.02 b	-0.04 b	-0.01 b	0.00 ab	-0.27 b	0.40 a	0.07 ab	-0.29 b	11	66590176	66593083	0.03	NA	0.03	1.31E-10

CCS	0.04 ab	-0.08 bc	0.01 ab	-0.01 abc	-0.13 bc	0.31 a	-0.11 bc	-0.50 c	11	66592821	66606019	-0.01	NA	0.03	2.67E-11
RBM4	-0.01 b	-0.09 b	0.00 b	0.07 ab	-0.30 b	0.40 a	-0.01 b	-0.29 b	11	66638617	66666682	0.10	NA	0.04	2.04E-11
C11orf80	0.12 b	0.06 b	-0.06 b	0.12 ab	0.14 ab	0.49 a	-0.07 b	-0.31 b	11	66744451	66843328	0.05	NA	0.04	2.06E-19
RCE1	0.09 b	0.10 b	-0.11 b	0.31 ab	0.07 b	0.69 a	0.10 b	-0.22 b	11	66842835	66846546	-0.03	NA	0.04	4.34E-40
PC	0.06 ab	-0.12 b	-0.02 b	0.03 ab	-0.07 b	0.43 a	0.05 ab	-0.12 b	11	66848233	66958376	0.06	NA	0.04	5.98E-12
LRFN4	0.19 ab	0.09 b	-0.09 b	0.01 b	0.21 ab	0.47 a	-0.01 b	-0.11 b	11	66856647	66860475	0.01	NA	0.04	8.26E-21
RHOD	-0.04 b	-0.01 ab	-0.01 b	0.13 ab	0.10 ab	0.30 a	-0.03 b	-0.28 b	11	67056818	67072013	0.02	NA	0.04	2.78E-06
KDM2A	0.03 b	-0.02 b	-0.06 b	0.20 b	-0.15 b	0.82 a	0.02 b	-0.40 b	11	67119269	67258087	0.08	NA	0.04	6.02E-46
GRK2	0.09 b	-0.05 b	-0.03 b	-0.11 b	-0.23 b	0.56 a	-0.11 b	-0.26 b	11	67266410	67286556	0.04	NA	0.04	1.20E-20
ANKRD13D	0.11 b	0.01 b	-0.08 b	0.17 b	0.11 b	0.75 a	-0.06 b	-0.29 b	11	67288547	67302485	0.03	NA	0.04	1.54E-39
SSH3	0.09 b	0.04 b	-0.07 b	0.21 ab	0.13 ab	0.63 a	-0.10 b	-0.34 b	11	67303448	67312607	0.08	NA	0.04	1.02E-29
RAD9A	0.18 b	0.04 bc	-0.09 bc	0.19 b	0.10 bc	0.76 a	-0.02 bc	-0.45 c	11	67317871	67398410	-0.03	NA	0.04	1.33E-47
POLD4	-0.02 bc	-0.06 bc	0.00 b	-0.17 bc	-0.37 bc	0.56 a	-0.13 bc	-0.49 c	11	67350777	67356972	0.03	NA	0.04	1.67E-25
PPP1CA	0.12 b	-0.01 bc	-0.06 bc	0.06 bc	-0.13 bc	0.75 a	-0.05 bc	-0.41 c	11	67398183	67421183	0.02	NA	0.04	1.86E-39
RPS6KB2	0.13 b	-0.01 b	-0.06 b	0.08 b	0.13 ab	0.61 a	0.04 b	-0.49 b	11	67428460	67435408	0.08	NA	0.04	7.68E-30
CORO1B	0.07 b	-0.08 bc	-0.02 bc	-0.03 bc	-0.42 bc	0.66 a	0.00 bc	-0.53 c	11	67435510	67443821	0.02	NA	0.04	1.20E-33
CABP4	-0.04 b	-0.10 b	-0.07 b	-0.04 b	-0.40 bc	1.64 a	-0.15 b	-1.06 c	11	67452406	67460313	NA	0.11	0.04	7.53E-214
TMEM134	-0.05 b	-0.10 b	-0.01 b	0.07 b	-0.35 b	0.70 a	-0.07 b	-0.43 b	11	67461710	67469272	-0.07	NA	0.04	2.53E-34
AIP	0.06 b	-0.04 bc	-0.02 b	-0.03 bc	-0.08 bc	0.54 a	-0.09 bc	-0.56 c	11	67483041	67491103	0.06	NA	0.04	2.70E-24
PITPNM1	-0.08 b	-0.10 b	-0.05 b	-0.02 b	-0.32 bc	1.51 a	-0.14 b	-1.03 c	11	67491768	67506263	0.09	-0.22	0.04	6.17E-181
CDK2AP2	0.02 b	0.01 b	-0.03 bc	0.00 bc	-0.18 bc	0.57 a	-0.03 bc	-0.58 c	11	67506497	67508649	-0.01	NA	0.04	5.03E-26
NDUFV1	0.06 b	0.00 b	-0.03 b	-0.04 bc	-0.06 bc	0.58 a	-0.08 bc	-0.54 c	11	67606852	67612535	-0.07	NA	0.04	3.38E-26
NUDT8	-0.14 b	-0.06 ab	0.02 ab	0.10 ab	0.02 ab	0.27 a	-0.01 ab	-0.33 b	11	67627938	67629930	0.04	NA	0.05	1.31E-07

ALDH3B2	0.01 b	-0.01 b	-0.05 b	0.25 ab	0.12 ab	0.54 a	-0.03 b	-0.08 b	11	67662162	67681200	0.01	NA	0.05	2.38E-18
UNC93B1	0.01 b	-0.08 b	-0.07 b	-0.02 b	-0.40 b	1.66 a	-0.15 b	-1.22 c	11	67991104	68004982	0.06	-0.01	0.04	6.81E-230
ALDH3B1	-0.01 b	-0.09 b	-0.07 b	-0.02 b	-0.37 b	1.72 a	-0.16 b	-1.28 c	11	68008578	68029282	NA	-0.05	0.05	6.74E-250
NDUFS8	0.05 b	0.00 b	-0.04 bc	-0.03 bc	-0.02 bc	0.70 a	-0.06 bc	-0.60 c	11	68030617	68036644	0.01	NA	0.05	1.45E-37
TCIRG1	0.00 b	-0.10 b	0.01 b	-0.07 b	0.03 ab	0.40 a	-0.17 b	-0.37 b	11	68039016	68050895	0.00	NA	0.04	1.32E-13
CHKA	0.07 ab	0.00 b	-0.02 b	-0.21 bc	-0.16 bc	0.41 a	0.06 ab	-0.66 c	11	68052859	68121444	-0.05	NA	0.04	1.82E-20
KMT5B	-0.01 b	-0.11 b	-0.01 b	0.15 b	-0.41 bc	0.74 a	0.00 b	-0.73 c	11	68154863	68213828	0.06	NA	0.04	9.19E-48
C11orf24	0.01 bc	-0.02 bc	-0.06 bc	0.07 bc	-0.02 bc	0.78 a	0.13 b	-0.54 c	11	68261335	68272001	0.03	NA	0.04	1.21E-44
LRP5	-0.05 b	-0.12 b	-0.05 b	-0.08 b	-0.36 b	1.64 a	-0.11 b	-1.35 c	11	68312609	68449275	0.00	-0.10	0.04	1.47E-233
PPP6R3	0.04 b	-0.06 b	-0.05 b	0.16 b	-0.23 bc	0.89 a	0.02 b	-0.68 c	11	68460731	68615334	0.06	NA	0.04	2.65E-61
TESMIN	0.07 b	0.01 b	-0.07 b	-0.05 b	-0.33 b	0.82 a	-0.01 b	-0.38 b	11	68707440	68751564	0.07	NA	0.04	3.03E-45
CPT1A	-0.03 b	-0.14 b	-0.09 b	-0.08 b	-0.03 b	1.85 a	-0.04 b	-1.04 c	11	68754620	68844410	0.07	-0.09	0.05	3.87E-270
MRPL21	0.02 b	-0.10 b	-0.11 b	0.01 b	-0.19 b	1.95 a	-0.06 b	-1.03 c	11	68891276	68903835	0.04	0.19	0.05	2.94E-303
IGHMBP2	-0.03 b	-0.11 b	-0.09 b	0.01 b	-0.15 bc	1.76 a	-0.12 b	-0.91 c	11	68903842	68940602	0.05	-0.32	0.05	1.02E-236
MRGPRD	-0.02 ab	-0.11 ab	-0.01 ab	0.36 a	-0.10 ab	0.35 a	-0.08 ab	0.05 ab	11	68980021	68980986	0.03	NA	0.05	1.44E-10
TPCN2	-0.04 b	-0.12 b	-0.10 b	0.08 b	-0.18 b	1.97 a	-0.06 b	-1.05 c	11	69048897	69162440	0.00	-0.16	0.04	0.00E+00
CCND1	0.01 b	-0.15 b	-0.12 b	-0.11 b	-0.20 bc	2.14 a	-0.12 b	-0.86 c	11	69641087	69654474	0.04	-0.02	0.05	0
ORAOV1	-0.01 b	-0.12 bc	-0.12 bc	-0.08 bc	-0.06 bc	2.00 a	-0.11 bc	-0.76 c	11	69653076	69675416	0.08	-0.19	0.05	2.27E-302
ANO1	-0.08 b	-0.14 b	-0.12 b	0.05 b	-0.02 b	2.12 a	-0.02 b	-0.82 c	11	70078302	70189528	0.01	-0.04	0.05	0
FADD	0.07 b	-0.05 bc	-0.11 bc	0.18 b	-0.04 bc	1.43 a	0.01 b	-0.59 c	11	70203163	70207390	-0.03	NA	0.05	1.82E-147
PPFIA1	0.01 b	-0.11 b	-0.14 b	0.17 b	-0.10 b	2.10 a	0.03 b	-0.86 c	11	70270700	70384403	-0.01	0.09	0.05	0
CTTN	0.02 b	-0.13 b	-0.13 b	0.08 b	-0.02 b	2.02 a	0.00 b	-0.86 c	11	70398404	70436584	0.03	0.04	0.05	0
SHANK2	-0.10 b	-0.09 b	-0.11 b	0.25 b	0.17 b	1.78 a	0.07 b	-0.84 c	11	70467856	71252577	0.04	-0.07	0.05	3.59E-244

DHCR7	0.13 b	0.08 b	-0.09 bc	0.00 bc	-0.21 bc	0.73 a	0.14 b	-0.41 c	11	71428193	71452868	0.01	NA	0.04	5.83E-43
NADSYN1	-0.09 b	-0.13 b	-0.06 b	0.08 b	-0.03 b	1.67 a	-0.09 b	-1.25 c	11	71453109	71524107	0.05	-0.19	0.04	1.60E-233
KRTAP5-7	-0.01 b	-0.04 b	-0.04 b	0.08 ab	-0.10 b	0.59 a	0.02 b	-0.13 b	11	71527267	71528674	0.03	NA	0.04	2.17E-20
KRTAP5-8	-0.06 b	-0.03 b	-0.03 b	0.07 ab	0.02 ab	0.50 a	-0.01 b	-0.03 b	11	71538025	71539207	0.00	NA	0.04	4.14E-14
KRTAP5-9	-0.09 b	-0.04 b	-0.01 b	0.06 ab	-0.09 b	0.45 a	-0.05 b	-0.22 b	11	71548418	71549553	-0.03	NA	0.04	4.90E-12
KRTAP5-10	-0.01 b	-0.04 b	-0.01 b	-0.10 b	-0.05 b	0.44 a	-0.06 b	-0.26 b	11	71565563	71566738	-0.04	NA	0.04	1.00E-11
FAM86C1	-0.02 b	0.04 b	-0.07 b	0.15 b	-0.25 bc	0.93 a	0.07 b	-0.75 c	11	71787510	71801236	0.02	NA	0.03	3.71E-70
RNF121	-0.05 b	-0.03 b	-0.03 b	0.18 b	-0.22 bc	0.79 a	-0.02 b	-0.94 c	11	71928701	71997597	-0.01	NA	0.03	6.34E-60
NUMA1	-0.11 b	-0.14 b	-0.02 b	-0.01 b	-0.45 b	1.55 a	-0.10 b	-1.71 c	11	72002864	72080693	0.00	-0.11	0.03	1.49E-246
LRTOMT	-0.12 b	-0.08 b	0.02 b	0.02 b	-0.40 bc	0.61 a	-0.11 b	-0.81 c	11	72080331	72110782	0.01	NA	0.03	4.42E-40
LAMTOR1	-0.10 b	-0.06 b	-0.01 b	0.08 b	-0.36 bc	0.75 a	0.01 b	-0.94 c	11	72085895	72103387	0.05	NA	0.03	1.23E-56
ANAPC15	-0.11 b	-0.03 b	0.02 b	0.02 b	-0.19 bc	0.46 a	-0.13 b	-0.72 c	11	72106378	72112780	0.00	NA	0.03	3.16E-25
INPPL1	0.16 b	-0.06 b	0.05 b	0.01 b	-0.13 bc	0.76 a	0.01 b	-0.75 c	11	72223701	72239105	0.00	NA	0.03	5.98E-51
CLPB	0.10 bc	0.14 b	-0.10 cd	0.14 bc	0.01 bcd	0.74 a	0.09 bc	-0.57 d	11	72292425	72434680	-0.04	NA	0.03	2.31E-50
ARAP1	-0.08 bc	-0.06 bc	0.01 b	-0.11 bc	-0.04 bc	0.59 a	-0.15 bc	-0.78 c	11	72685069	72793599	0.05	NA	0.03	7.45E-36
STARD10	0.03 b	-0.05 b	-0.01 b	-0.09 bc	-0.23 bc	0.66 a	-0.09 bc	-0.67 c	11	72754729	72794168	0.07	NA	0.03	8.25E-37
ATG16L2	-0.06 b	-0.03 b	0.02 b	-0.01 b	0.11 ab	0.34 a	-0.28 b	0.52 b	11	72814308	72843674	0.00	NA	0.03	1.68E-15
FCHSD2	-0.14 bc	-0.08 b	0.03 b	0.02 b	-0.09 bc	0.48 a	-0.17 bc	-0.72 c	11	72836745	73142261	0.13	NA	0.03	3.54E-28
ARHGEF17	-0.16 bc	-0.10 bc	0.05 ab	-0.11 bc	-0.14 bc	0.31 a	-0.08 abc	-0.48 c	11	73308289	73369091	0.06	NA	0.03	4.06E-15
RELT	0.03 b	0.05 b	-0.05 b	0.11 ab	0.10 ab	0.41 a	-0.01 b	-0.25 b	11	73376264	73397474	0.08	NA	0.03	1.60E-11
RAB6A	0.07 b	0.00 b	-0.06 b	0.15 b	-0.30 bc	0.73 a	0.14 b	-0.68 c	11	73675638	73761137	-0.09	NA	0.03	5.61E-46
MRPL48	0.06 b	0.06 b	-0.05 b	0.04 b	-0.18 bc	0.55 a	0.16 ab	-0.78 c	11	73787316	73865133	0.00	NA	0.03	5.02E-35
COA4	0.07 b	0.03 b	-0.05 b	0.03 b	-0.24 bc	0.68 a	0.11 b	-0.94 c	11	73872667	73876988	0.00	NA	0.03	9.29E-51

PAAF1	0.01 b	0.07 b	-0.03 b	-0.08 b	-0.16 bc	0.54 a	0.00 b	-0.89 c	11	73876699	73931124	0.04	NA	0.03	3.25E-36
UCP3	-0.16 b	0.00 ab	0.01 ab	0.03 ab	-0.15 ab	0.30 a	-0.01 ab	-0.46 b	11	74000281	74009435	0.06	NA	0.03	8.15E-11
C2CD3	-0.07 b	-0.02 b	-0.04 b	0.15 b	-0.32 bc	0.74 a	0.14 b	-0.69 c	11	74012714	74171210	0.00	NA	0.03	8.74E-47
PPME1	0.07 b	0.05 b	-0.09 b	0.12 b	-0.25 bc	0.88 a	0.19 b	-0.66 c	11	74171099	74254703	0.05	NA	0.03	4.14E-64
LIPT2	-0.02 b	0.03 b	-0.04 b	0.01 b	-0.45 bc	0.52 a	0.21 ab	-0.65 c	11	74491712	74493733	-0.09	NA	0.03	1.00E-28
POLD3	-0.03 b	0.02 b	-0.06 b	0.32 ab	-0.12 bc	0.56 a	0.22 ab	-0.56 c	11	74493851	74669117	0.14	NA	0.03	5.18E-31
RNF169	-0.06 bc	-0.05 bc	-0.03 bc	0.25 ab	-0.26 bc	0.63 a	0.11 b	-0.46 c	11	74748868	74842413	0.01	NA	0.03	1.43E-30
XRRA1	-0.01 b	-0.08 b	0.00 b	0.01 b	-0.42 bc	0.66 a	0.05 b	-0.91 c	11	74807739	74949200	0.05	NA	0.03	3.25E-47
SPCS2	0.04 b	-0.01 b	-0.01 b	0.02 b	-0.24 bc	0.55 a	-0.06 b	-1.04 c	11	74949247	74979031	0.00	NA	0.03	4.36E-44
NEU3	0.01 b	-0.02 b	-0.02 b	0.04 ab	-0.20 b	0.45 a	0.03 ab	-0.43 b	11	74988134	75018893	0.10	NA	0.03	4.20E-15
ARRB1	-0.08 b	-0.06 b	-0.02 b	-0.11 b	-0.42 b	1.31 a	-0.02 b	-1.77 c	11	75264182	75351705	NA	-0.03	0.03	9.70E-200
RPS3	-0.05 b	-0.03 b	0.02 b	-0.08 b	-0.39 bc	0.41 a	-0.05 b	-0.91 c	11	75399486	75422280	0.06	NA	0.03	3.08E-30
UVRAG	-0.10 b	0.02 b	-0.04 b	0.24 ab	-0.48 bc	0.69 a	0.11 b	-0.72 c	11	75815167	76143195	0.09	NA	0.03	1.14E-44
THAP12	0.01 b	-0.02 b	-0.02 b	0.04 b	-0.44 bc	0.60 a	0.13 b	-0.83 c	11	76349956	76380971	0.02	NA	0.02	4.51E-39
EMSY	-0.06 bc	-0.07 bc	-0.03 b	0.15 b	-0.31 bc	0.69 a	0.08 b	-0.53 c	11	76444923	76553025	0.02	NA	0.03	4.76E-36
LRRC32	-0.06 b	-0.05 b	-0.04 b	-0.05 b	-0.45 b	1.34 a	-0.03 b	-1.51 c	11	76657524	76670747	NA	-0.06	0.03	4.02E-180
TSKU	-0.11 bc	-0.12 bc	0.02 bc	0.02 abc	-0.06 bc	0.46 a	0.09 ab	-0.53 c	11	76782251	76798154	0.08	NA	0.03	6.54E-21
ACER3	-0.06 bc	0.00 b	-0.04 b	0.14 b	-0.15 bc	0.66 a	0.11 b	-0.55 c	11	76860867	77026797	0.03	NA	0.03	8.76E-34
MYO7A	-0.05 b	-0.05 b	-0.05 b	-0.03 b	-0.39 b	1.44 a	-0.03 b	-1.40 c	11	77128264	77215239	NA	-0.07	0.03	3.28E-189
PAK1	0.00 b	-0.02 b	-0.04 b	0.13 b	-0.55 c	0.70 a	0.06 b	-0.55 c	11	77321707	77474635	0.03	NA	0.03	2.36E-38
CLNS1A	0.08 b	0.04 b	-0.08 b	0.14 b	-0.42 bc	0.82 a	0.14 b	-0.76 c	11	77514936	77637805	0.03	NA	0.03	7.70E-61
AQP11	-0.01 b	-0.03 b	0.00 b	-0.16 b	-0.36 b	0.40 a	-0.02 b	-0.45 b	11	77589391	77610355	0.01	NA	0.03	7.05E-14
RSF1	-0.05 b	-0.06 b	-0.05 b	-0.02 b	-0.43 b	1.40 a	0.02 b	-1.26 c	11	77659996	77821017	0.01	0.26	0.03	1.19E-171

AAMDC	-0.06 b	-0.03 b	0.00 b	-0.07 bc	-0.27 bc	0.51 a	-0.07 bc	-0.68 c	11	77821109	77918432	-0.08	NA	0.03	5.25E-26
INTS4	-0.05 b	-0.01 b	-0.05 b	0.14 b	-0.36 bc	0.99 a	0.06 b	-0.85 c	11	77878720	77994678	0.00	NA	0.03	1.20E-79
NDUFC2	0.03 b	-0.05 b	-0.04 b	-0.07 b	-0.33 bc	0.87 a	0.01 b	-0.83 c	11	78068304	78080219	0.00	NA	0.03	5.68E-64
ALG8	0.06 b	0.07 b	-0.09 b	0.02 b	-0.24 bc	1.05 a	0.05 b	-0.84 c	11	78100936	78139660	0.07	NA	0.03	1.48E-91
KCTD21	0.04 b	-0.08 b	-0.03 b	0.07 b	-0.20 bc	0.80 a	-0.03 b	-0.74 c	11	78171249	78188822	-0.05	NA	0.03	2.07E-52
USP35	0.03 b	-0.04 b	0.05 b	-0.11 b	-0.31 b	1.34 a	-0.07 b	-1.33 c	11	78188812	78214711	0.04	-0.07	0.03	1.49E-165
NARS2	0.04 b	0.02 b	-0.03 b	0.09 ab	-0.48 bc	0.60 a	0.04 b	-0.75 c	11	78435961	78574874	-0.08	NA	0.03	4.23E-36
CCDC77	0.11 a	0.03 a	-0.05 a	0.34 a	-0.24 a	0.01 a	0.10 a	0.22 a	12	389273	442645	0.08	NA	0.03	1.02E-05
WNK1	0.09 ab	-0.01 ab	-0.05 b	0.15 ab	-0.34 b	0.28 a	0.08 ab	0.11 ab	12	752593	911452	0.07	NA	0.03	1.27E-06
ADIPOR2	0.12 ab	0.00 ab	-0.06 b	0.24 ab	-0.40 b	0.31 a	0.12 ab	0.14 ab	12	1688574	1788678	0.00	NA	0.03	8.02E-11
DCP1B	-0.10 a	-0.03 a	0.05 a	0.00 a	-0.64 a	-0.08 a	-0.14 a	-0.08 a	12	1946054	2004535	0.06	NA	0.03	1.78E-05
FKBP4	0.21 ab	-0.01 bc	-0.08 c	0.14 abc	-0.38 c	0.38 a	0.12 abc	0.37 ab	12	2794953	2805423	0.01	NA	0.03	3.77E-20
RHNO1	0.22 a	0.02 ab	-0.07 ab	0.24 a	-0.46 ab	0.21 ab	0.13 ab	0.05 ab	12	2876258	2889523	0.02	NA	0.03	4.58E-11
TULP3	0.14 a	0.01 a	-0.05 a	0.30 a	-0.26 a	0.15 a	0.10 a	0.05 a	12	2877223	2941140	0.00	NA	0.03	4.54E-06
TSPAN9	-0.08 a	-0.10 a	0.06 a	-0.07 a	-0.36 a	-0.01 a	-0.28 a	-0.06 a	12	3077355	3286564	-0.06	NA	0.03	4.44E-07
PARP11	-0.09 a	-0.06 a	0.05 a	-0.19 a	-0.68 a	0.02 a	-0.14 a	-0.11 a	12	3791047	3873448	0.07	NA	0.03	1.08E-06
CCND2	-0.09 ab	0.00 a	0.08 a	-0.31 ab	-0.28 ab	-0.38 b	-0.22 ab	-0.20 ab	12	4273772	4305350	-0.05	NA	0.03	5.54E-17
RAD51AP1	0.25 ab	0.13 ab	-0.13 b	0.41 a	0.08 ab	0.25 ab	0.24 ab	0.26 ab	12	4538798	4560048	0.01	NA	0.03	1.09E-27
DYRK4	-0.16 a	-0.03 a	0.06 a	-0.16 a	-0.48 a	-0.10 a	-0.15 a	-0.19 a	12	4562204	4615302	0.07	NA	0.03	8.96E-08
AKAP3	-0.15 ab	-0.11 ab	0.09 a	-0.20 ab	-0.34 b	-0.24 b	-0.10 ab	0.01 ab	12	4615508	4649047	0.01	NA	0.03	4.76E-12
TNFRSF1A	-0.02 a	-0.02 a	0.00 a	0.03 a	-0.86 b	0.22 a	-0.05 ab	0.06 a	12	6328757	6342114	0.04	-0.12	0.03	2.66E-07
LTBR	0.14 a	0.07 a	-0.06 a	0.25 a	-0.25 a	0.21 a	0.08 a	-0.02 a	12	6375045	6391571	0.04	NA	0.03	6.41E-08
NCAPD2	0.11 ab	0.10 ab	-0.10 ab	0.35 a	-0.16 ab	0.24 a	0.24 a	0.24 a	12	6493356	6531955	0.03	NA	0.03	1.92E-16

GAPDH	0.16 a	0.11 a	-0.07 a	0.20 a	-0.14 a	0.07 a	0.08 a	0.24 a	12	6533927	6538374	0.01	NA	0.03	1.14E-08
NOP2	0.15 ab	0.13 ab	-0.09 b	0.17 ab	-0.19 b	0.24 a	0.19 ab	0.12 ab	12	6556863	6568691	-0.09	NA	0.03	1.03E-13
ING4	-0.04 a	-0.06 a	0.04 a	0.25 a	-0.52 a	-0.18 a	-0.02 a	-0.04 a	12	6650280	6663148	0.02	NA	0.03	2.19E-05
ZNF384	0.01 a	0.05 a	-0.05 a	0.22 a	-0.46 a	0.25 a	0.14 a	-0.02 a	12	6666477	6689572	0.06	NA	0.03	3.32E-07
COPS7A	0.00 a	0.04 a	-0.04 a	0.21 a	-0.52 a	0.17 a	0.06 a	0.22 a	12	6723741	6731875	0.06	NA	0.03	7.81E-06
MLF2	0.09 a	0.02 a	-0.04 a	0.06 a	-0.45 a	0.23 a	0.10 a	0.10 a	12	6747996	6767475	0.03	NA	0.03	9.65E-06
CDCA3	0.27 a	0.17 ab	-0.14 b	0.31 a	0.23 ab	0.27 a	0.25 a	0.31 a	12	6844793	6852066	-0.04	NA	0.03	1.25E-33
USP5	0.06 a	0.09 a	-0.06 a	0.17 a	-0.36 a	0.15 a	0.13 a	0.27 a	12	6852128	6866632	-0.03	NA	0.03	9.67E-08
TPI1	0.17 a	0.13 a	-0.08 ab	0.25 a	-0.23 ab	0.00 ab	0.12 ab	0.25 a	12	6867119	6870948	0.03	NA	0.03	1.25E-10
ATN1	0.03 a	-0.03 a	-0.02 a	0.08 a	-0.53 a	0.25 a	0.04 a	0.10 a	12	6924463	6942321	0.01	NA	0.03	3.65E-05
C12orf57	-0.31 b	-0.22 b	0.11 a	-0.12 ab	-0.56 b	0.02 ab	-0.09 ab	-0.07 ab	12	6942978	6946003	0.03	NA	0.03	4.00E-24
SCARNA12	0.14 a	0.12 a	-0.07 a	0.18 a	-0.13 a	0.15 a	0.06 a	0.01 a	12	6967337	6967606	0.01	NA	0.03	1.25E-07
EMG1	0.18 a	0.11 a	-0.06 a	0.09 a	-0.19 a	0.08 a	0.08 a	-0.01 a	12	6970893	6979941	0.03	NA	0.03	1.05E-06
LPCAT3	-0.07 b	0.05 ab	-0.04 b	0.13 ab	-0.37 b	0.31 a	0.12 ab	-0.03 ab	12	6976186	7018510	0.01	NA	0.03	1.34E-07
NECAP1	0.11 ab	-0.05 ab	-0.01 ab	0.00 ab	-0.67 b	0.16 a	-0.02 ab	0.11 ab	12	8076939	8097859	0.00	NA	0.03	2.30E-05
CLEC4A	0.01 ab	0.01 ab	-0.01 ab	-0.03 ab	-0.79 b	0.21 a	-0.04 ab	0.07 ab	12	8123632	8138607	NA	0.05	0.03	4.06E-06
DDX12P	0.17 a	0.09 ab	-0.10 ab	0.33 a	0.10 ab	0.26 a	0.13 ab	0.14 ab	12	9418673	9448229	0.02	NA	0.03	4.89E-15
GABARAPL1	-0.11 a	-0.08 a	0.05 a	-0.02 a	-0.65 a	-0.13 a	0.11 a	0.00 a	12	10212458	10223130	0.02	NA	0.03	1.48E-06
MAGOHB	0.19 a	0.16 a	-0.10 ab	0.12 ab	-0.16 ab	0.20 a	0.06 ab	0.26 a	12	10604190	10613623	0.01	NA	0.03	1.96E-15
LOH12CR2	-0.13 a	-0.03 a	0.05 a	0.13 a	-0.59 a	-0.12 a	-0.03 a	-0.22 a	12	12355406	12357067	0.02	NA	0.03	1.54E-06
BORCS5	-0.01 a	0.04 a	-0.04 a	0.21 a	-0.61 a	0.13 a	0.16 a	0.20 a	12	12357079	12469694	0.07	NA	0.03	2.00E-06
CREBL2	-0.10 a	-0.11 a	0.06 a	-0.06 a	-0.70 a	0.08 a	-0.12 a	-0.17 a	12	12611827	12645108	0.00	NA	0.03	1.86E-08
GPR19	0.14 ab	0.14 ab	-0.11 b	0.13 ab	0.21 ab	0.23 a	0.21 ab	0.37 a	12	12660891	12696207	-0.06	NA	0.03	5.50E-19

DDX47	0.05 a	0.02 a	-0.04 a	0.22 a	-0.54 a	0.12 a	0.20 a	0.16 a	12	12813316	12829981	-0.01	NA	0.03	3.30E-06
FAM234B	-0.04 b	0.01 b	-0.05 b	0.12 ab	-0.11 b	0.41 a	0.07 ab	0.13 ab	12	13044284	13142521	0.04	NA	0.03	2.99E-10
WBP11	0.12 ab	0.01 ab	-0.05 ab	0.15 ab	-0.60 b	0.23 a	0.10 ab	0.19 ab	12	14784579	14803540	0.02	NA	0.03	1.13E-08
STRAP	0.15 a	0.08 a	-0.06 a	0.15 a	-0.51 a	0.10 a	0.17 a	0.17 a	12	15882391	15903478	0.04	NA	0.03	1.35E-08
DERA	0.01 a	0.05 a	-0.03 a	0.37 a	-0.29 a	-0.04 a	0.22 a	-0.01 a	12	15911172	16037282	0.07	NA	0.03	2.47E-05
RECQL	0.11 a	0.04 a	-0.05 a	0.30 a	-0.18 a	0.09 a	0.15 a	0.12 a	12	21468911	21501669	0.02	NA	0.02	1.33E-05
GOLT1B	0.18 a	0.04 ab	-0.07 ab	0.32 a	-0.16 ab	0.13 ab	0.18 ab	0.14 ab	12	21501781	21518408	0.03	NA	0.02	6.73E-10
CMAS	0.27 a	0.06 ab	-0.07 b	0.23 ab	-0.45 b	0.03 ab	0.07 ab	0.19 ab	12	22046174	22065674	0.01	NA	0.02	1.98E-11
ETFRF1	-0.09 a	-0.10 a	0.07 a	0.17 a	-0.55 a	-0.12 a	-0.20 a	-0.12 a	12	25195216	25209645	0.02	NA	0.02	6.68E-09
KRAS	0.12 a	0.02 a	-0.06 a	0.31 a	-0.26 a	0.15 a	0.21 a	0.15 a	12	25204789	25250936	0.08	NA	0.02	3.64E-08
INTS13	0.12 a	0.12 a	-0.07 a	0.31 a	-0.21 a	0.01 a	0.18 a	0.06 a	12	26905181	26938326	0.00	NA	0.02	3.16E-08
MED21	0.04 a	0.02 a	-0.03 a	0.36 a	-0.51 a	0.05 a	0.13 a	0.07 a	12	27022546	27066343	0.03	NA	0.02	2.47E-05
DDX11	0.08 a	0.09 a	-0.07 a	0.36 a	-0.15 a	0.17 a	0.11 a	0.17 a	12	31073845	31104791	0.04	NA	0.02	2.73E-09
H3F3C	0.09 ab	0.00 ab	-0.03 ab	0.07 ab	-0.71 b	0.20 a	0.04 ab	0.05 ab	12	31791185	31792241	NA	0.04	0.02	4.35E-06
DNM1L	0.13 a	0.03 a	-0.07 a	0.38 a	-0.34 a	0.19 a	0.16 a	0.11 a	12	32679200	32745650	0.04	NA	0.02	3.68E-10
YARS2	0.16 a	0.09 a	-0.06 a	0.27 a	-0.22 a	0.05 a	0.06 a	0.07 a	12	32727490	32755902	0.02	NA	0.02	2.30E-06
ALG10	0.01 ab	0.02 ab	-0.05 ab	0.36 a	-0.25 ab	0.26 a	0.16 ab	0.05 ab	12	34022281	34029694	0.00	NA	0.02	1.38E-08
HOXC13	0.04 ab	0.22 ab	-0.14 b	0.43 a	0.38 ab	0.38 ab	0.17 ab	0.39 a	12	53938765	53946544	0.01	NA	NA	7.99E-37
HOXC11	-0.05 ab	0.21 a	-0.10 ab	0.33 a	-0.14 ab	0.28 a	0.19 ab	0.21 ab	12	53973126	53977643	0.03	NA	NA	1.84E-21
HOXC8	-0.03 ab	0.22 ab	-0.10 b	0.26 ab	-0.06 ab	0.31 a	0.06 ab	0.15 ab	12	54009106	54012362	0.00	NA	NA	3.51E-19
GPR132	-0.22 ab	0.09 ab	-0.04 ab	0.19 a	0.35 a	0.18 a	0.18 ab	0.17 ab	14	105049389	105065445	NA	-0.01	NA	4.84E-10
WASH3P	-0.25 ab	0.22 a	-0.04 ab	0.24 a	0.36 a	-0.02 ab	0.01 ab	0.07 ab	15	101961603	101976543	0.02	NA	NA	4.07E-14
MYO1C	-0.15 b	0.12 ab	-0.03 ab	-0.06 ab	-0.27 b	0.26 a	0.06 ab	-0.04 ab	17	1464098	1492812	NA	-0.07	NA	9.07E-08

NTN1	0.02 ab	0.05 a	0.03 a	-0.09 ab	-0.24 ab	-0.18 ab	-0.37 b	-0.06 ab	17	9021542	9244000	NA	0.02	NA	1.54E-07
TRIM16L	-0.21 b	0.08 ab	-0.05 b	0.15 ab	0.26 ab	0.29 a	0.43 a	-0.04 ab	17	18697998	18736118	0.02	NA	NA	1.67E-17
ANKRD13B	0.06 ab	0.25 a	-0.12 ab	0.27 a	0.31 a	0.20 a	0.10 ab	0.27 a	17	29589769	29614761	0.04	NA	NA	3.07E-23
SPACA3	-0.38 b	-0.01 ab	0.02 ab	0.12 ab	-0.08 ab	0.18 a	0.12 a	0.05 ab	17	32970376	32997877	0.02	NA	NA	4.45E-13
SCARNA17	-0.27 ab	0.12 a	0.01 ab	0.05 ab	0.14 a	-0.03 ab	0.04 ab	-0.22 ab	18	49814133	49814276	0.02	NA	NA	6.98E-08
ABCA7	0.02 ab	0.19 a	-0.08 ab	0.21 a	0.15 ab	0.21 a	0.06 ab	0.00 ab	19	1040101	1065572	NA	-0.02	NA	8.46E-12
EPS15L1	-0.16 bc	-0.20 c	0.05 b	0.01 bc	-0.47 c	0.35 a	0.02 bc	-0.28 c	19	16355239	16472085	NA	-0.03	NA	6.44E-19
CEACAM8	-0.32 ab	0.02 ab	0.01 ab	0.09 ab	0.33 a	0.13 a	0.19 a	-0.02 ab	19	42580241	42595055	0.01	NA	NA	5.35E-10
JOSD2	-0.01 b	0.00 b	-0.04 b	0.13 ab	-0.09 b	0.41 a	-0.01 b	-0.04 b	19	50505998	50511353	NA	-0.04	NA	2.56E-09
FKBP1AP1	-0.28 ab	0.19 a	-0.02 ab	0.05 ab	0.29 a	0.07 ab	0.03 ab	-0.08 ab	19	57826461	57826778	0.00	NA	NA	3.41E-11
DEFB126	-0.10 ab	0.10 ab	-0.07 ab	0.34 a	0.13 ab	0.31 a	0.19 ab	0.05 ab	20	142369	145751	0.07	NA	0.03	7.72E-13
C20orf96	-0.58 b	0.36 a	0.00 b	0.02 ab	0.08 ab	-0.08 b	-0.07 b	-0.12 b	20	270863	290778	0.01	NA	0.04	2.44E-47
ZCCHC3	-0.86 c	0.52 a	-0.04 b	0.18 ab	0.36 ab	-0.03 b	0.20 ab	0.09 ab	20	296968	300321	0.08	NA	0.04	1.21E-111
SOX12	-0.53 c	0.35 a	-0.07 b	0.09 ab	0.35 ab	0.24 ab	0.24 ab	0.17 ab	20	325595	330224	-0.08	NA	0.04	5.60E-51
NRSN2	-0.54 c	0.28 a	-0.03 b	0.05 ab	0.26 ab	0.12 ab	0.17 ab	0.17 ab	20	346782	359660	0.03	NA	0.04	1.29E-38
TRIB3	-0.14 ab	0.33 a	-0.10 ab	-0.12 ab	0.44 a	0.16 ab	0.26 a	0.25 ab	20	362835	397559	0.03	NA	0.03	4.92E-31
RBCK1	-0.80 c	0.62 a	-0.06 b	0.05 b	0.36 ab	-0.01 b	0.13 b	0.09 b	20	407498	430966	0.02	NA	0.04	1.42E-122
TBC1D20	-0.91 c	0.51 a	-0.04 b	0.11 b	0.21 ab	0.14 b	0.17 ab	0.09 b	20	435480	462553	0.00	NA	0.04	6.61E-117
CSNK2A1	-0.77 d	0.58 a	-0.10 c	0.24 abc	0.26 abc	0.21 bc	0.28 ab	0.27 abc	20	473591	543821	0.00	NA	0.04	1.93E-121
SRXN1	-0.44 b	0.39 a	-0.10 b	0.08 ab	0.31 ab	0.25 ab	0.42 a	0.21 ab	20	646615	653370	0.01	NA	0.04	5.15E-57
SLC52A3	-0.24 c	0.22 a	-0.04 bc	0.06 abc	0.16 abc	0.17 ab	0.07 abc	-0.14 bc	20	760080	776015	0.02	NA	0.04	1.34E-13
FAM110A	-0.51 b	0.39 a	-0.07 a	0.13 a	0.49 a	0.22 a	0.10 a	0.23 a	20	833715	857463	0.00	NA	0.04	3.39E-53
PSMF1	-1.05 c	0.67 a	-0.07 b	0.11 b	0.44 ab	0.20 b	0.27 b	0.11 b	20	1113263	1189415	0.06	NA	0.04	3.66E-186

TMEM74B	-0.12 ab	0.29 a	-0.05 ab	0.07 ab	0.32 a	-0.08 ab	0.06 ab	-0.10 ab	20	1180561	1185415	-0.04	NA	0.04	2.59E-15
C20orf202	-0.18 ab	0.21 a	-0.02 ab	-0.20 ab	0.21 a	-0.05 ab	0.08 ab	-0.05 ab	20	1203454	1208274	0.03	NA	0.04	5.42E-09
SNPH	-1.77 e	1.12 a	-0.09 d	0.04 cd	0.78 ab	0.21 bc	0.24 bc	0.26 bc	20	1266316	1309328	0.00	-0.04	0.04	0
SDCBP2	-0.33 b	0.17 a	0.00 ab	0.15 ab	0.14 ab	-0.10 ab	0.10 ab	0.01 ab	20	1309909	1329239	0.03	NA	0.04	3.23E-13
FKBP1A	-0.76 b	0.44 a	-0.03 a	0.05 a	0.48 a	0.03 a	0.22 a	0.11 a	20	1368978	1393172	0.00	NA	0.04	1.65E-83
NSFL1C	-1.02 c	0.64 a	-0.08 b	0.27 ab	0.56 ab	0.15 b	0.22 b	0.28 ab	20	1442162	1473842	-0.02	NA	0.04	1.93E-175
SIRPA	-0.25 b	0.13 a	0.00 ab	0.12 ab	0.02 ab	-0.04 ab	-0.01 ab	-0.11 ab	20	1894167	1940592	-0.01	NA	0.04	8.68E-07
STK35	-0.63 c	0.56 a	-0.13 b	0.26 ab	0.31 ab	0.33 a	0.26 ab	0.39 a	20	2101611	2177038	0.06	NA	0.04	4.34E-109
TGM6	-1.82 e	1.13 a	-0.09 d	0.02 cd	0.76 ab	0.26 bc	0.25 bc	0.28 bc	20	2380908	2432753	NA	-0.05	0.04	0
SNRPB	-0.61 c	0.58 a	-0.12 b	0.22 ab	0.64 a	0.25 ab	0.24 ab	0.30 ab	20	2461634	2470853	-0.01	NA	0.04	8.01E-109
ZNF343	-0.81 c	0.57 a	-0.09 b	0.21 b	0.34 ab	0.24 b	0.21 b	0.16 b	20	2481817	2524702	0.08	NA	0.04	8.30E-123
TMC2	-0.30 b	0.18 a	0.00 ab	0.00 ab	0.10 ab	0.03 ab	-0.10 ab	-0.02 ab	20	2536607	2641784	-0.01	NA	0.04	3.89E-11
NOP56	-0.64 c	0.55 a	-0.10 b	0.08 b	0.49 ab	0.15 b	0.22 ab	0.29 ab	20	2652145	2658393	0.07	NA	0.04	3.45E-96
IDH3B	-0.91 c	0.62 a	-0.06 b	0.18 b	0.42 ab	0.00 b	0.19 b	0.27 ab	20	2658395	2664219	-0.04	NA	0.04	1.62E-143
EBF4	-0.40 b	0.08 a	0.03 a	-0.13 ab	-0.20 ab	0.07 a	-0.07 ab	0.17 a	20	2692878	2760108	0.01	NA	0.04	1.39E-14
PCED1A	-0.64 b	0.46 a	-0.03 a	0.00 a	0.52 a	0.03 a	0.02 a	-0.15 ab	20	2835314	2841190	0.04	NA	0.04	6.69E-69
VPS16	-0.92 c	0.61 a	-0.08 b	0.16 b	0.56 ab	0.17 b	0.20 b	0.25 ab	20	2840703	2866732	0.02	NA	0.04	2.78E-148
PTPRA	-0.88 c	0.52 a	-0.04 b	0.10 b	0.27 ab	0.18 b	0.11 b	0.12 b	20	2864184	3039076	0.08	NA	0.04	6.66E-115
MRPS26	-0.76 c	0.57 a	-0.06 b	0.05 b	0.42 ab	0.08 b	0.15 b	0.07 b	20	3045945	3048254	0.05	NA	0.04	3.16E-106
OXT	-1.81 e	1.15 a	-0.10 d	0.04 cd	0.76 ab	0.25 bc	0.24 bc	0.28 bc	20	3071620	3072517	NA	0.01	0.04	0
UBOX5	-0.90 c	0.51 a	-0.03 b	-0.02 b	0.31 ab	0.22 ab	0.11 b	-0.05 b	20	3107573	3160196	0.00	NA	0.04	1.43E-115
FASTKD5	-0.78 c	0.55 a	-0.07 b	0.16 ab	0.40 ab	0.12 b	0.20 ab	0.14 ab	20	3146519	3159897	0.06	NA	0.04	1.38E-107
DDRGK1	-0.84 c	0.52 a	-0.01 b	-0.13 b	0.14 ab	0.02 b	-0.02 b	-0.02 b	20	3190350	3204685	0.06	NA	0.04	3.47E-104

ITPA	-0.76 c	0.51 a	-0.05 b	0.18 ab	0.39 ab	0.02 b	0.17 ab	0.09 ab	20	3208868	3223870	0.02	NA	0.04	1.49E-94
SLC4A11	-0.37 b	0.14 a	0.02 ab	-0.09 ab	0.04 ab	0.03 ab	-0.02 ab	-0.13 ab	20	3227417	3239190	0.08	NA	0.04	5.10E-13
C20orf194	-0.56 ab	0.12 a	0.02 a	0.12 a	0.16 a	0.15 a	0.06 a	-0.07 ab	20	3249305	3407625	0.02	NA	0.04	2.36E-28
ATRN	-0.74 c	0.36 a	-0.05 b	0.17 ab	0.17 ab	0.32 a	0.31 a	0.13 ab	20	3471040	3651122	0.03	NA	0.04	2.33E-78
ADAM33	-1.83 e	1.13 a	-0.09 d	0.03 cd	0.74 ab	0.24 bc	0.24 bc	0.33 bc	20	3667965	3682246	NA	-0.01	0.04	0
HSPA12B	-1.74 d	1.11 a	-0.10 c	0.08 bc	0.73 ab	0.22 b	0.26 b	0.37 b	20	3732667	3753111	NA	-0.06	0.04	0
C20orf27	-1.71 e	1.13 a	-0.11 d	0.07 cd	0.81 ab	0.23 bc	0.25 bc	0.32 bc	20	3753508	3768387	0.05	0.05	0.04	0
SPEF1	-0.36 b	0.23 a	0.00 b	0.03 ab	0.11 ab	-0.04 b	-0.03 b	-0.05 b	20	3777504	3781448	0.00	NA	0.04	1.58E-16
CENPB	-0.88 c	0.57 a	-0.04 b	0.07 b	0.37 ab	0.06 b	0.06 b	0.16 ab	20	3783851	3786690	0.04	NA	0.04	2.18E-121
CDC25B	-0.27 ab	0.45 a	-0.10 ab	0.18 ab	0.49 a	0.07 ab	0.14 ab	0.15 ab	20	3786772	3806121	-0.05	NA	0.04	4.42E-46
AP5S1	-0.87 c	0.60 a	-0.05 b	-0.01 b	0.47 ab	0.03 b	0.10 b	0.04 b	20	3820524	3828837	0.04	NA	0.04	6.14E-128
MAVS	-0.73 d	0.52 a	-0.06 c	0.00 bc	0.17 abc	0.24 ab	0.17 abc	0.02 bc	20	3846799	3876123	0.00	NA	0.04	1.68E-95
PANK2	-0.94 c	0.49 a	-0.03 b	0.19 ab	0.38 ab	0.01 b	0.19 ab	0.18 ab	20	3888839	3929882	0.06	NA	0.04	1.77E-118
RNF24	-0.53 b	0.30 a	-0.05 b	0.15 ab	0.24 ab	0.21 ab	0.22 ab	0.06 ab	20	3927309	4015582	0.01	NA	0.04	6.54E-42
SMOX	-1.79 e	1.14 a	-0.10 d	-0.01 cd	0.75 ab	0.23 bc	0.23 bc	0.30 bc	20	4120980	4187747	0.02	-0.13	0.04	0
PRNP	-0.46 ab	0.17 a	0.01 ab	0.13 a	0.31 a	-0.04 ab	0.04 a	0.01 ab	20	4686236	4701590	0.01	NA	0.04	6.22E-21
PRND	-1.79 e	1.13 a	-0.10 d	0.04 cd	0.77 ab	0.27 bc	0.21 bc	0.33 bc	20	4721910	4728460	NA	-0.07	0.04	0
SLC23A2	-0.54 b	0.18 a	-0.01 ab	0.16 ab	0.16 ab	0.22 a	0.04 ab	0.06 ab	20	4852356	5010293	0.02	NA	0.04	2.17E-30
TMEM230	-0.87 c	0.58 a	-0.05 b	0.19 ab	0.18 ab	0.13 b	0.07 b	0.04 b	20	5068232	5113103	0.05	NA	0.04	5.73E-124
PCNA	-0.48 b	0.50 a	-0.12 a	0.31 a	0.58 a	0.17 a	0.22 a	0.35 a	20	5114953	5126626	0.00	NA	0.04	3.74E-78
CDS2	-0.82 c	0.51 a	-0.06 b	0.11 b	0.42 ab	0.28 ab	0.12 b	0.09 b	20	5126786	5197887	0.00	NA	0.04	1.04E-108
PROKR2	-1.82 e	1.15 a	-0.10 d	0.05 cd	0.76 ab	0.27 bc	0.22 bc	0.27 bc	20	5302040	5314369	NA	-0.03	0.04	0
GPCPD1	-0.48 c	0.32 a	0.00 b	0.11 ab	-0.09 bc	-0.11 bc	-0.17 bc	0.05 ab	20	5544404	5611026	-0.04	NA	0.04	2.83E-35

C20orf196	-0.63 c	0.41 a	0.01 b	-0.08 b	-0.09 bc	-0.15 bc	-0.13 bc	0.02 ab	20	5750393	5863912	0.04	NA	0.04	1.42E-59
CHGB	-0.24 ab	0.04 ab	0.05 a	-0.20 ab	-0.24 ab	-0.12 ab	-0.12 ab	0.05 a	20	5911430	5925361	0.00	NA	0.04	1.59E-07
TRMT6	-0.65 c	0.63 a	-0.13 b	0.47 a	0.66 a	0.14 ab	0.23 a	0.37 a	20	5937235	5950558	0.05	NA	0.04	2.67E-127
MCM8	-0.45 b	0.48 a	-0.14 a	0.39 a	0.53 a	0.29 a	0.28 a	0.33 a	20	5950652	5998977	0.08	NA	0.04	3.12E-81
CRLS1	-0.74 c	0.56 a	-0.04 b	0.09 b	0.16 ab	-0.05 b	0.13 b	-0.06 b	20	6006090	6040053	-0.04	NA	0.04	3.67E-99
LRRN4	-1.82 e	1.13 a	-0.09 d	0.02 cd	0.73 ab	0.25 bc	0.21 bc	0.25 bc	20	6040778	6054049	0.00	-0.23	0.04	0
FERMT1	-1.71 d	1.08 a	-0.09 c	0.08 bc	0.70 ab	0.22 b	0.22 b	0.21 bc	20	6074845	6123544	0.05	0.29	0.04	0
BMP2	-0.31 b	0.14 a	0.03 ab	0.10 ab	0.07 ab	-0.23 b	-0.06 ab	-0.02 ab	20	6767664	6780280	-0.07	NA	0.04	6.35E-13
TMX4	-0.61 c	0.28 a	0.02 b	0.10 ab	-0.17 bc	-0.14 b	0.02 ab	0.03 ab	20	7977348	8019829	0.11	NA	0.04	1.08E-42
PLCB1	-0.37 b	0.16 a	0.00 a	0.17 a	-0.16 ab	-0.01 ab	0.08 a	-0.03 ab	20	8077251	8968360	0.02	NA	0.04	3.30E-14
PLCB4	-0.16 a	0.10 a	0.04 a	-0.06 a	-0.68 a	-0.22 a	-0.17 a	-0.09 a	20	9068763	9481242	0.05	NA	0.04	6.38E-11
ANKEF1	-0.48 c	0.41 a	-0.08 bc	0.28 ab	0.17 abc	0.30 a	0.10 abc	0.06 abc	20	9986126	10058303	0.07	NA	0.04	3.15E-56
SNAP25	-0.22 ab	0.05 a	0.04 a	0.12 a	-0.04 ab	-0.08 ab	-0.21 ab	-0.07 ab	20	10218830	10307418	0.01	NA	0.04	3.55E-06
MKKS	-0.92 c	0.59 a	-0.06 b	0.20 ab	0.30 ab	0.09 b	0.19 b	0.22 ab	20	10401009	10434222	0.05	NA	0.04	3.32E-139
SLX4IP	-0.43 c	0.31 a	-0.07 bc	0.29 ab	0.22 abc	0.32 a	0.18 abc	0.08 abc	20	10435303	10636829	0.01	NA	0.04	4.54E-41
JAG1	-0.42 c	0.22 a	-0.02 bc	0.19 ab	0.16 abc	0.13 abc	-0.07 bc	0.13 abc	20	10637684	10674107	0.00	NA	0.04	1.37E-22
BTBD3	-0.58 c	0.30 a	-0.02 bc	0.28 ab	0.14 abc	-0.03 bc	0.16 ab	0.12 abc	20	11890723	11926609	0.06	NA	0.03	2.76E-44
SPTLC3	-0.44 b	0.10 a	0.06 a	0.16 a	-0.20 ab	-0.24 ab	-0.16 ab	-0.03 ab	20	13008979	13169103	0.01	NA	0.03	4.76E-23
TASP1	-0.75 c	0.49 a	-0.05 b	0.26 ab	0.29 ab	0.08 b	0.11 b	0.08 b	20	13389392	13638940	-0.08	NA	0.03	2.45E-89
ESF1	-0.58 c	0.49 a	-0.10 b	0.32 ab	0.45 ab	0.20 ab	0.18 ab	0.21 ab	20	13714322	13784886	0.05	NA	0.03	1.21E-80
NDUFAF5	-0.81 c	0.59 a	-0.06 b	0.21 ab	0.45 ab	0.00 b	0.08 b	0.18 ab	20	13784950	13821582	0.07	NA	0.03	2.31E-117
MACROD2	-0.36 b	0.10 a	0.06 ab	0.06 ab	-0.09 ab	-0.22 ab	-0.12 ab	-0.20 ab	20	13995369	16053197	0.01	NA	0.03	4.52E-17
FLRT3	-0.35 b	0.15 a	0.02 ab	0.20 a	0.06 ab	-0.11 ab	-0.15 ab	0.14 ab	20	14322988	14337616	0.02	NA	0.03	8.51E-15

KIF16B	-0.53 c	0.39 a	-0.07 bc	0.22 ab	0.18 abc	0.25 a	0.13 abc	0.18 ab	20	16272104	16573434	0.04	NA	0.03	1.10E-55
SNRPB2	-0.74 c	0.67 a	-0.10 b	0.26 ab	0.62 ab	0.00 b	0.17 b	0.24 ab	20	16729961	16742563	-0.03	NA	0.03	1.35E-133
BFSP1	-0.37 ac	0.35 a	-0.07 abc	0.26 ab	0.40 a	-0.03 abc	0.23 ab	0.26 ab	20	17493905	17569220	0.04	NA	0.03	3.55E-38
DSTN	-0.66 c	0.36 a	-0.02 b	0.13 ab	0.18 ab	0.04 ab	0.12 ab	0.08 ab	20	17569863	17609919	0.12	NA	0.03	1.05E-57
RRBP1	-1.72 e	1.09 a	-0.09 d	0.07 cd	0.78 ab	0.22 bc	0.20 bc	0.26 bc	20	17613678	17682295	0.02	-0.01	0.03	0
SNX5	-0.79 c	0.46 a	-0.02 b	0.06 b	0.30 ab	0.07 b	0.11 ab	-0.13 b	20	17941597	17968980	0.00	NA	0.03	7.95E-87
SNORD17	-0.42 b	0.25 a	-0.04 ab	0.10 a	0.41 a	0.18 a	0.18 a	0.01 ab	20	17962710	17962946	0.03	NA	0.03	1.50E-28
MGME1	-0.68 c	0.58 a	-0.13 b	0.41 a	0.71 a	0.25 ab	0.31 a	0.25 ab	20	17968913	17991122	-0.03	NA	0.03	9.72E-124
ZNF133	-0.81 c	0.55 a	-0.07 b	0.22 ab	0.53 ab	0.11 b	0.15 b	0.15 ab	20	18288283	18316996	0.07	NA	0.03	5.22E-113
DZANK1	-0.51 c	0.42 a	-0.04 bc	0.14 ab	0.07 abc	0.01 bc	-0.10 bc	0.09 ab	20	18383367	18467281	0.00	NA	0.03	5.46E-50
POLR3F	-0.75 c	0.60 a	-0.09 b	0.32 ab	0.45 ab	0.09 b	0.15 b	0.23 ab	20	18467127	18484643	0.08	NA	0.03	1.19E-117
RBBP9	-0.74 c	0.43 a	-0.02 b	0.25 ab	0.10 ab	-0.03 b	0.02 b	0.06 ab	20	18486540	18497243	0.02	NA	0.03	2.23E-76
SEC23B	-0.63 c	0.37 a	-0.03 bc	0.06 ab	0.02 abc	0.14 ab	0.08 ab	0.08 ab	20	18507493	18561415	0.00	NA	0.03	1.19E-55
SMIM26	-0.90 c	0.58 a	-0.03 b	0.09 b	0.26 ab	-0.02 b	0.05 b	0.01 b	20	18567347	18569563	0.00	NA	0.03	5.27E-126
DTD1	-0.58 c	0.46 a	-0.04 b	0.12 ab	0.26 ab	-0.18 b	0.16 ab	0.19 ab	20	18567453	18763917	-0.07	NA	0.03	1.65E-66
LINC00652	-0.16 b	0.30 a	-0.04 b	-0.04 b	-0.05 b	-0.07 b	-0.11 b	0.07 ab	20	18786065	18794335	-0.07	NA	0.03	1.24E-15
SLC24A3	-0.19 ab	0.09 a	0.03 ab	-0.04 ab	-0.02 ab	-0.01 ab	-0.30 b	-0.15 ab	20	19212646	19722937	-0.07	NA	0.03	2.92E-07
RIN2	-0.46 b	0.31 a	-0.04 b	0.15 ab	0.23 ab	0.05 ab	0.11 ab	0.07 ab	20	19886521	20002457	0.03	NA	0.03	1.21E-32
NAA20	-0.61 c	0.60 a	-0.13 b	0.25 b	0.53 ab	0.27 b	0.22 b	0.22 b	20	20017116	20033655	-0.04	NA	0.03	3.61E-110
CRNKL1	-0.73 c	0.50 a	-0.07 b	0.23 ab	0.15 ab	0.20 ab	0.15 ab	0.15 ab	20	20034368	20056046	0.05	NA	0.03	5.95E-93
DEFB127	-0.10 ab	0.10 ab	-0.07 ab	0.34 a	0.13 ab	0.31 a	0.19 ab	0.05 ab	20	17786826.02	17952273.32	0.01	NA	0.03	-7.80E-11
C20orf97	-0.58 b	0.36 a	0.00 b	0.02 ab	0.08 ab	-0.08 b	-0.07 b	-0.12 b	20	17990961.07	18157997.76	0.01	NA	0.03	-7.88E-11
ZCCHC4	-0.86 c	0.52 a	-0.04 b	0.18 ab	0.36 ab	-0.03 b	0.20 ab	0.09 ab	20	18195096.11	18363722.19	0.00	NA	0.03	-7.96E-11

SOX13	-0.53 c	0.35 a	-0.07 b	0.09 ab	0.35 ab	0.24 ab	0.24 ab	0.17 ab	20	18399231.16	18569446.62	0.00	NA	0.03	-8.03E-11
NRSN3	-0.54 c	0.28 a	-0.03 b	0.05 ab	0.26 ab	0.12 ab	0.17 ab	0.17 ab	20	18603366.21	18775171.06	0.00	NA	0.03	-8.11E-11
TRIB4	-0.14 ab	0.33 a	-0.10 ab	-0.12 ab	0.44 a	0.16 ab	0.26 a	0.25 ab	20	18807501.26	18980895.49	0.00	NA	0.03	-8.19E-11
RBCK2	-0.80 c	0.62 a	-0.06 b	0.05 b	0.36 ab	-0.01 b	0.13 b	0.09 b	20	19011636.3	19186619.92	0.00	NA	0.03	-8.27E-11
TBC1D21	-0.91 c	0.51 a	-0.04 b	0.11 b	0.21 ab	0.14 b	0.17 ab	0.09 b	20	19215771.35	19392344.35	0.00	NA	0.03	-8.34E-11
CSNK2A2	-0.77 d	0.58 a	-0.10 c	0.24 abc	0.26 abc	0.21 bc	0.28 ab	0.27 abc	20	19419906.4	19598068.79	0.00	NA	0.03	-8.42E-11
SRXN2	-0.44 b	0.39 a	-0.10 b	0.08 ab	0.31 ab	0.25 ab	0.42 a	0.21 ab	20	19624041.44	19803793.22	0.00	NA	0.03	-8.50E-11
SLC52A4	-0.24 c	0.22 a	-0.04 bc	0.06 abc	0.16 abc	0.17 ab	0.07 abc	-0.14 bc	20	19828176.49	20009517.65	0.00	NA	0.03	-8.57E-11
FAM110A	-0.51 b	0.39 a	-0.07 a	0.13 a	0.49 a	0.22 a	0.10 a	0.23 a	20	20032311.54	20215242.08	0.00	NA	0.03	-8.65E-11
PSMF2	-1.05 c	0.67 a	-0.07 b	0.11 b	0.44 ab	0.20 b	0.27 b	0.11 b	20	20236446.59	20420966.52	0.00	NA	0.03	-8.73E-11
TMEM74B	-0.12 ab	0.29 a	-0.05 ab	0.07 ab	0.32 a	-0.08 ab	0.06 ab	-0.10 ab	20	20440581.63	20626690.95	0.00	NA	0.03	-8.81E-11
C20orf203	-0.18 ab	0.21 a	-0.02 ab	-0.20 ab	0.21 a	-0.05 ab	0.08 ab	-0.05 ab	20	20644716.68	20832415.38	0.00	NA	0.03	-8.88E-11
SNPH	-1.77 e	1.12 a	-0.09 d	0.04 cd	0.78 ab	0.21 bc	0.24 bc	0.26 bc	20	20848851.73	21038139.82	-0.01	-0.04	0.03	-9E-11
SDCBP3	-0.33 b	0.17 a	0.00 ab	0.15 ab	0.14 ab	-0.10 ab	0.10 ab	0.01 ab	20	21052986.78	21243864.25	-0.01	NA	0.03	-9.04E-11
FKBP1A	-0.76 b	0.44 a	-0.03 a	0.05 a	0.48 a	0.03 a	0.22 a	0.11 a	20	21257121.82	21449588.68	-0.01	NA	0.03	-9.12E-11
NSFL1C	-1.02 c	0.64 a	-0.08 b	0.27 ab	0.56 ab	0.15 b	0.22 b	0.28 ab	20	21461256.87	21655313.11	-0.01	NA	0.03	-9.19E-11
SIRPA	-0.25 b	0.13 a	0.00 ab	0.12 ab	0.02 ab	-0.04 ab	-0.01 ab	-0.11 ab	20	21665391.92	21861037.55	-0.01	NA	0.03	-9.27E-11
MUC6	-0.11 b	0.15 ab	-0.06 b	0.43 a	0.17 ab	0.10 ab	-0.04 ab	0.32 ab	*	1012821	1043528	NA	0.00	NA	1.99E-12
KAT14	-0.83 c	0.50 a	-0.03 b	0.04 b	0.20 ab	0.08 b	0.10 b	0.14 ab	NA	NA	NA	NA	NA	NA	4.33E-101
LOC653566	-0.02 b	-0.03 b	-0.01 b	0.05 b	-0.29 bc	0.67 a	0.03 b	-1.04 c	NA	NA	NA	NA	NA	NA	2.59E-53
LOC100270804	-0.42 b	0.37 a	-0.04 b	0.13 ab	0.28 ab	0.03 b	-0.04 b	-0.05 b	NA	NA	NA	NA	NA	NA	2.85E-36
LOC100130987	0.01 b	-0.10 b	-0.01 b	0.10 ab	-0.27 b	0.54 a	-0.03 b	-0.49 b	NA	NA	NA	NA	NA	NA	2.12E-23
ProSAPiP1	-0.35 b	0.27 a	0.00 b	0.09 ab	-0.10 b	-0.16 b	0.06 ab	-0.25 b	NA	NA	NA	NA	NA	NA	9.78E-23

LOC645332	-0.11 bc	0.00 b	0.00 b	-0.08 bc	-0.40 bc	0.50 a	-0.06 bc	-0.53 c	NA	NA	NA	NA	NA	NA	2.62E-22
LOC100289673	-0.12 ab	0.12 ab	-0.07 ab	0.27 a	0.50 a	0.24 a	0.16 ab	0.23 ab	NA	NA	NA	NA	NA	NA	3.90E-14
LOC730101	-0.06 ab	-0.08 ab	0.06 ab	0.28 a	0.23 ab	-0.10 ab	-0.47 b	-0.15 ab	NA	NA	NA	NA	NA	NA	4.63E-14
ATPGD1	-0.04 b	0.09 ab	-0.07 b	0.32 ab	0.27 ab	0.36 a	0.02 ab	0.09 ab	NA	NA	NA	NA	NA	NA	1.63E-13
WASH5P	-0.25 ab	0.21 a	-0.03 ab	0.23 a	0.17 ab	-0.04 ab	0.01 ab	0.04 ab	NA	NA	NA	NA	NA	NA	3.19E-12
C20orf46	-0.06 ab	0.17 a	-0.07 ab	0.29 a	-0.04 ab	0.19 a	0.10 ab	0.21 a	NA	NA	NA	NA	NA	NA	7.38E-12
C19orf22	-0.08 b	0.01 b	-0.04 b	0.30 ab	-0.05 b	0.38 a	0.00 b	-0.07 b	NA	NA	NA	NA	NA	NA	2.20E-10
SUV420H1	-0.13 b	-0.11 b	0.04 ab	-0.06 ab	-0.39 b	0.23 a	-0.01 ab	-0.38 b	NA	NA	NA	NA	NA	NA	2.32E-10
LINC01512	-0.02 ab	-0.01 ab	0.04 a	-0.02 ab	0.02 ab	-0.02 ab	-0.53 b	0.04 a	NA	NA	NA	NA	NA	NA	2.49E-10
LOC149837	-0.22 b	0.15 a	0.02 ab	-0.16 ab	-0.14 ab	0.00 ab	-0.14 ab	-0.16 ab	NA	NA	NA	NA	NA	NA	3.95E-08
SINHCAF	0.08 a	0.09 a	-0.04 a	0.40 a	-0.37 a	-0.11 a	0.17 a	0.05 a	NA	NA	NA	NA	NA	NA	6.33E-08
LOC100134868	-0.21 b	0.17 a	-0.01 ab	0.13 ab	0.10 ab	-0.10 ab	0.01 ab	-0.02 ab	NA	NA	NA	NA	NA	NA	2.18E-07
LOC642846	0.11 a	0.05 a	-0.07 a	0.25 a	0.10 a	0.18 a	0.11 a	0.11 a	NA	NA	NA	NA	NA	NA	1.25E-06
KIAA1026	0.10 a	-0.03 a	0.03 a	0.09 a	-0.03 a	-0.22 a	-0.29 a	0.01 a	NA	NA	NA	NA	NA	NA	2.81E-06
LOC374443	-0.09 b	-0.07 b	0.00 b	0.05 ab	-0.28 b	0.31 a	0.00 b	-0.02 b	NA	NA	NA	NA	NA	NA	6.53E-06

*CHR_HSCHR11_2_CTG1

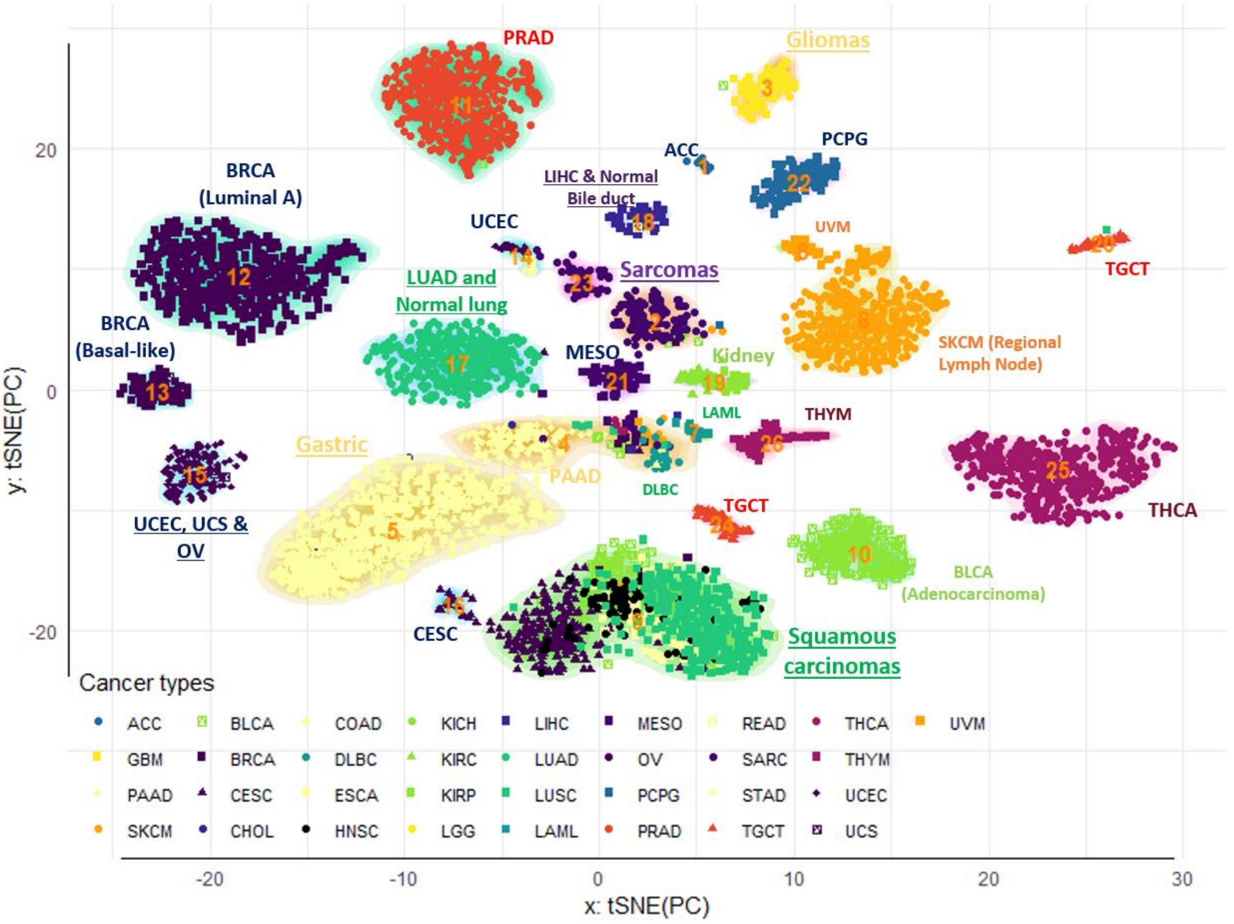

Figure S1: Pan-cancer clustering of tumor samples (no tissue effects correction, no sparsity constrains on the features activities). Tumor clusters were obtained by sequential application of tSNE and DBSCAN algorithm for 5,408 samples from 33 cancer types. The contours reflect cluster membership, and the points' colors and shapes represent similar anatomical site and cancer type, respectively. The two dimensional tSNE projection was obtained from the first 50 principal axes of the extended omic matrix, after removing the first two. Extended omic matrix contained appended values of gene expression, DNA methylation, and copy number variant intensity. Integers represent individual clusters. Clusters were also annotated in terms of their most enriched histological/molecular subtypes.

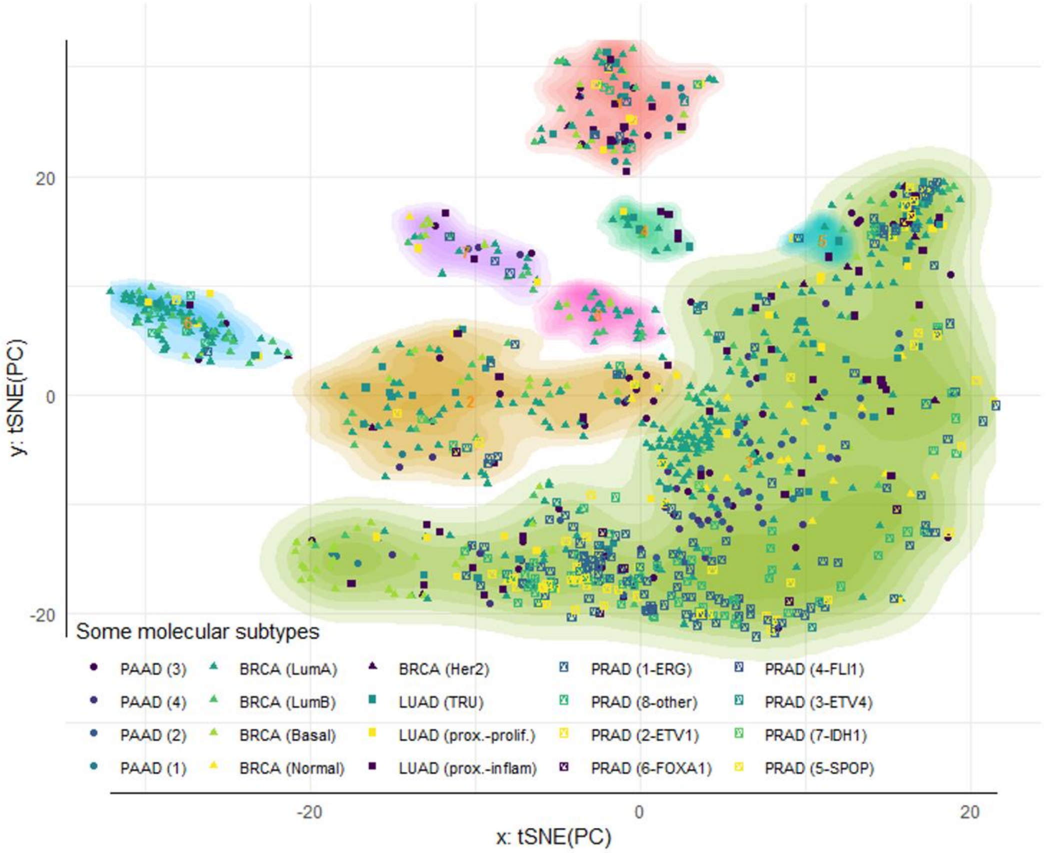

Figure S2: Re-classification of tumors after removing tissue effects does not agree with previously reported molecular subtypes.

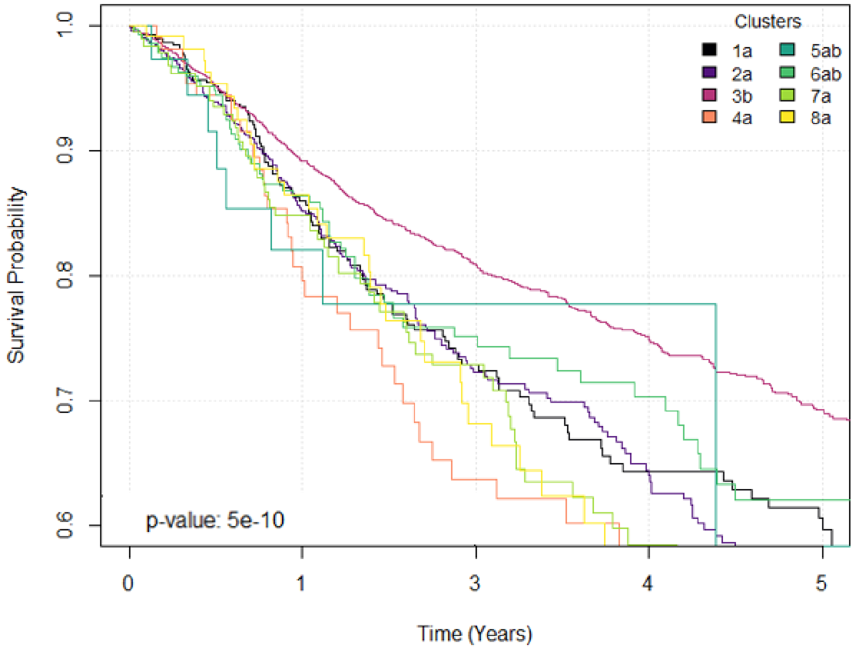

Figure S3: Survival curves by pan-cancer tumor clusters. The figure show Kaplan-Meier curves highlighting the survival probability by time in years for each cluster. Log-rank tests were performed to determined significant differences between curves. The legend shows the results of multiple comparison between survival curves. Statistical differences are represented with different letters.

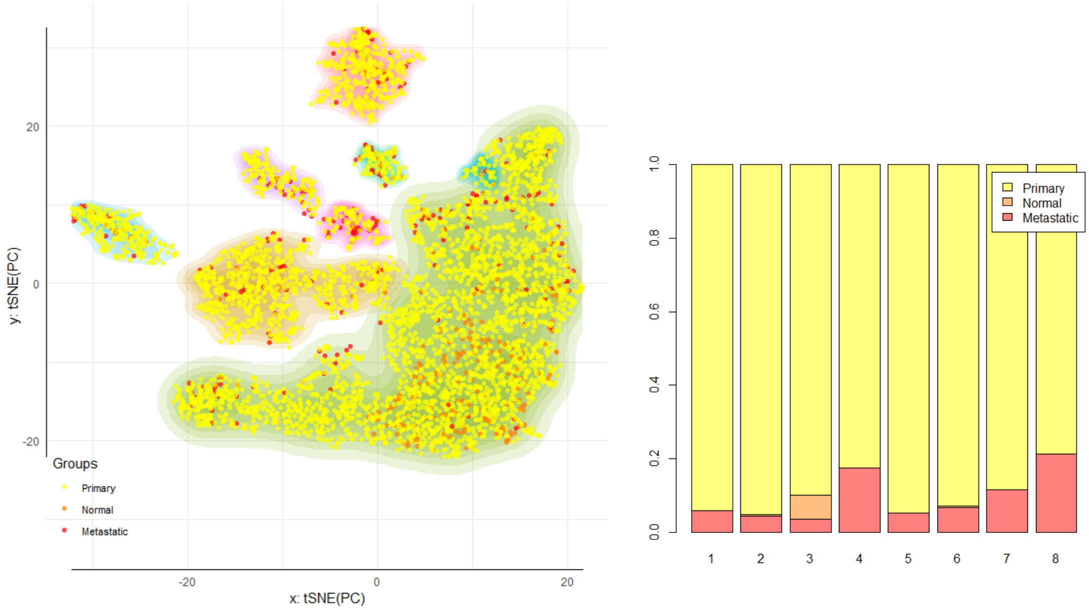

Figure S4: Re-classification of pan-cancer tumors after removing tissue effects reveals differences in sample type composition. The relative position and number of primary, normal, and metastatic tissue samples is shown. The figure at the left shows the samples location by clusters. The figure at the right shows the relative proportion of sample types by cluster.

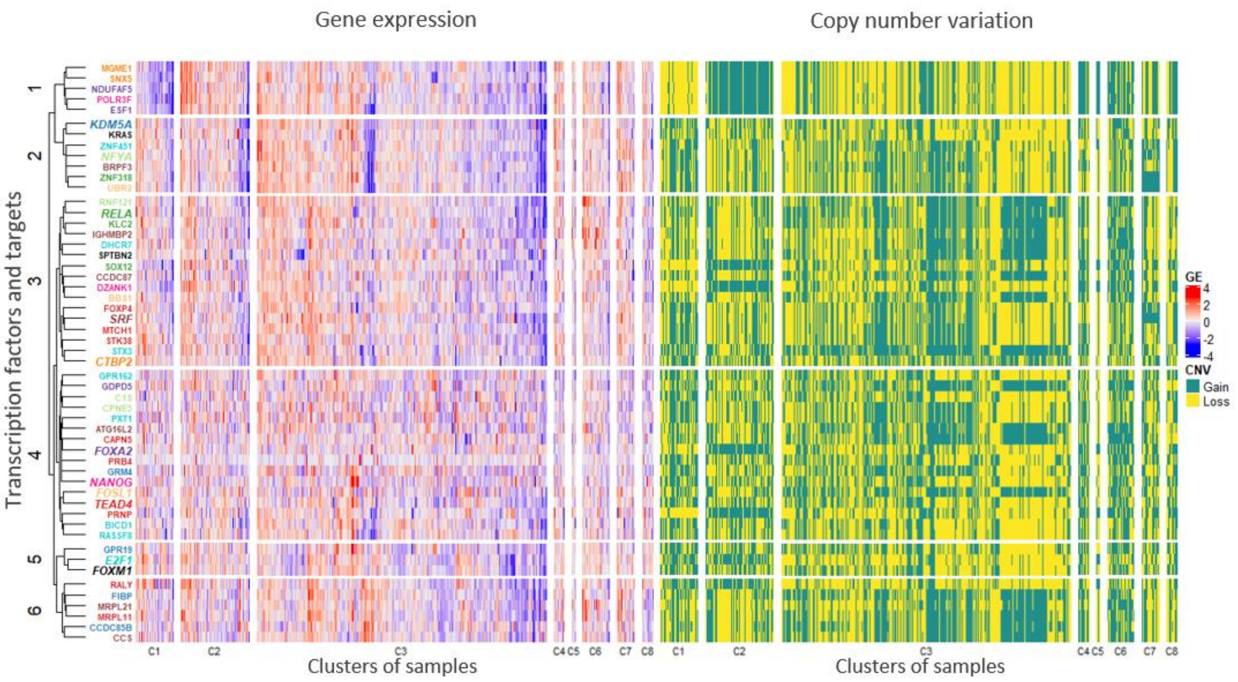

Figure S5: Relationship between gene expression and copy number variation for transcription factors and their targets within the set of selected features. The expression and copy number variation by gene is shown by cluster (C1-8). The colors by gene name represent groups defined by different transcription factors and their targets (e.g. black represents the group of *FOXM1* and its targets *KRAS* and *SPTBN2*). TFs names are shown with italic and larger font size. The number at the left of the dendogram represent grouping of genes based on k-means clustering.
